# CENP-A is a potential prognostic biomarker and correlated with immune infiltration levels in glioma patients

**DOI:** 10.3389/fgene.2022.931222

**Published:** 2022-08-29

**Authors:** Yuan Yang, Mengyun Duan, Yunfei Zha, Zijun Wu

**Affiliations:** ^1^ Department of Radiology, Renmin Hospital of Wuhan University, Wuhan, China; ^2^ Health Science Center, Department of Medical Imaging, Yangtze University, Jingzhou, China

**Keywords:** CENP-A, glioma, prognosis, biomarker, microenvironment

## Abstract

**Background:** Centromeric protein A (*CENP-A*), an essential protein involved in chromosomal segregation during cell division, is associated with several cancer types. However, its role in gliomas remains unclear. This study examined the clinical and prognostic significance of *CENP-A* in gliomas.

**Methods:** Data of patients with glioma were collected from the Cancer Genome Atlas. Logistic regression, the Kruskal–Wallis test, and the Wilcoxon signed-rank test were performed to assess the relationship between *CENP-A* expression and clinicopathological parameters. The Cox regression model and Kaplan–Meier curve were used to analyze the association between *CENP-A* and survival outcomes. A prognostic nomogram was constructed based on Cox multivariate analysis. Gene set enrichment analysis (GSEA) was conducted to identify key *CENP-A*-related pathways and biological processes.

**Results:** *CENP-A* was upregulated in glioma samples. Increased *CENP-A* levels were significantly associated with the world health organization (WHO) grade [Odds ratio (OR) = 49.88 (23.52–129.06) for grade 4 vs. grades 2 and 3], primary therapy outcome [OR = 2.44 (1.64–3.68) for progressive disease (PD) and stable disease (SD) vs. partial response (PR) and complete response (CR)], isocitrate dehydrogenase (IDH) status [OR = 13.76 (9.25–20.96) for wild-type vs. mutant], 1p/19q co-deletion [OR = 5.91 (3.95–9.06) for no codeletion vs. co-deletion], and age [OR = 4.02 (2.68–6.18) for > 60 vs. ≤ 60]. Elevated *CENP-A* expression was correlated with shorter overall survival in both univariate [hazard ratio (HR): 5.422; 95% confidence interval (CI): 4.044–7.271; *p* < 0.001] and multivariate analyses (HR: 1.967; 95% CI: 1.280–3.025; *p* < 0.002). GSEA showed enrichment of numerous cell cycle-and tumor-related pathways in the *CENP-A* high expression phenotype. The calibration plot and C-index indicated the favorable performance of our nomogram for prognostic prediction in patients with glioma.

**Conclusion:** We propose a role for *CENP-A* in glioma progression and its potential as a biomarker for glioma diagnosis and prognosis.

## Introduction

Gliomas are among the most lethal cancers and are characterized by invasive growth within the central nervous system. The intratumoral heterogeneity of gliomas and the intrinsic structure of contribute to tumor progression and treatment resistance (Jackson et al., 2019). Despite current multimodal therapies, including surgical resection and postoperative chemoradiotherapy, the prognosis of gliomas, especially high-grade gliomas, remains low with a median overall survival (OS) of 15 months (Weller et al., 2015). Owing to advances in the molecular genetics of gliomas in the past decade, the diagnostic classification, treatment development, and prognosis monitoring of gliomas have improved (Reifenberger et al., 2017). The novel glioma classification system integrates molecular biomarkers with classic histological features to define glioma entities (Wesseling and Capper, 2018). Additionally, preclinical and clinical studies have explored emerging pharmacological and immunotherapeutic strategies. Predictive molecular profiling has been proposed to guide individualized therapy in patients with glioma (Bi et al., 2020). However, further studies are required to investigate glioma biomarkers and therapeutic targets.

Centromeric factors are increasingly shown to be involved in tumor pathogenesis and have been proposed as potential therapeutic targets or prognostic markers (Filipescu et al., 2017). Centromeric protein A (CENP-A) is a histone H3-like protein that is enriched at active centromeres and serves as an epigenetic mark of centromere identity (Hoffmann et al., 2020). *CENP-A* regulates centromere integrity and chromosome segregation during cell division, and its overexpression leads to ectopic *CENP-A* deposition causing consequent defects in chromosome segregation (Lacoste et al., 2014). Accordingly, mislocalization of *CENP-A* resulting from its overexpression contributes to chromosomal instability and aneuploidy (Shrestha et al., 2021), which have long been recognized as hallmarks of tumor growth, malignant progression, and treatment resistance (Zhang et al., 2016; Sansregret et al., 2018). Recent studies have indicated that *CENP-A* overexpression induces chromosomal instability in cancer cells (Amato et al., 2009; Quevedo et al., 2020). Additionally, increased *CENP-A* expression is implicated in malignant progression (Sun et al., 2016) and correlates with poor prognosis in cancers (Zhang et al., 2020a; Saha et al., 2020; Xu et al., 2020), including breast (Rajput et al., 2011), lung (Wu et al., 2012; Liu et al., 2018), and hepatic carcinoma (Zhang et al., 2020b). *CENP-A* downregulation induces cell cycle arrest and cell death in hepatoma and lung carcinoma (Li et al., 2011; Wu et al., 2014). In patients with high-grade glioblastoma (GBM), increased *CENP-A* expression is associated with short OS(Stangeland et al., 2015; Chen et al., 2020). However, although *CENP-A* overexpression was proposed as a common feature of numerous cancer types (Li et al., 2019; Qi et al., 2019), its role in gliomas is unclear. The prognostic value of *CENP-A* in gliomas including GBM and low-grade gliomas remains to be investigated. In particular, the association between *CENP-A* expression and clinicopathological features of patients with glioma, as well as the detailed molecular mechanism of CENP-A in gliomas, have not been reported yet.

In the present study, we explored The Cancer Genome Atlas (TCGA) database to obtain glioma RNA-sequencing data and performed a series of bioinformatic analyses to comprehensively investigate *CENP-A* expression patterns and its prognostic significance in gliomas. We compared *CENP-A* expression among patients with glioma and healthy individuals, and analyzed the association of *CENP-A* mRNA expression with parameters in clinical data. To determine the effects of *CENP-A* on glioma prognosis, we performed survival analyses using *CENP-A* expression and clinicopathological features in the Cox regression model and developed a nomogram to predict glioma prognosis. We also verified the expression pattern and role of *CENP-A* at the mRNA level in The Chinese Glioma Genome Atlas (CGGA) cohort. To highlight the genes and functional pathways closely correlated with *CENP-A* expression, enrichment analysis was performed, including gene ontology (GO), Kyoto Encyclopedia of Genes and Genomes (KEGG), and gene set enrichment analysis (GSEA). Our study investigated the role of *CENP-A* in gliomas and discussed the possible *CENP-A-*related immune mechanisms involved in the pathogenesis of glioma.

## Materials and methods

### Data sources and pre-processing

We obtained publicly available RNA-seq and clinicopathological data of 696 glioma patient samples from TCGA and data of normal brain samples from the GTEx database. For subsequent analyses, all gene expression profiles were processed using Toil software (Vivian et al., 2017) and normalized as values in transcripts per million reads (TPM). The relevant clinical information of patients included age, gender, world health organization (WHO) grade, histological diagnosis, status of molecular markers, and follow-up outcomes. Our study conforms to the publication requirements of TCGA. For further validation, glioma data from the Chinese cohorts were obtained from CGGA datasets.

### Differentially expressed genes analysis

*CENP-A* expression in patients across TCGA was statistically ranked by median value and defined as high and low expression groups. Differentially expressed genes (DEGs) between the high- and low-*CENP-A* expression groups were identified *via* entry of expression profiles (HTseq-Counts) into the DESeq2 R package (Love et al., 2014). Genes with | log2 fold change (FC) | > 2.0 and an adjusted *p* < 0.01 were included to obtain statistically significant differences.

### Metascape enrichment analysis

Metascape is a well-maintained online portal for comprehensive gene list analyses and interpretations. Herein, enrichment analysis of pathways and biological processes was performed for *CENP-A*-specific DEGs using Metascape (Zhou et al., 2019). Only conditions with an enrichment factor > 1.5, minimum count of 3, and *p* < 0.01 were considered significant. To further explore DEGs, protein-protein interaction (PPI) networks were modeled by importing the data from three databases, BioGrid, OminiPath, and InWeb_IM, into Metascape along with the Molecular Complex Detection (MCODE) algorithm, in which the tightly connected PPI network components were identified.

### Gene set enrichment analysis

To explore the underlying functional or pathway differences between high- and low-*CENP-A* groups, GSEA was conducted using the R package clusterProfiler (3.14.3) (Yu et al., 2012). For each analysis, gene cluster random permutations were performed 1,000 times. The terms with |NES| > 1, adjusted *p* < 0.05, and FDR *q* value < 0.25 were interpreted as statistically significant differences between the groups.

### Analysis of immune infiltration and its correlation with centromeric protein A expression

By adopting the single-sample GSEA (ssGSEA) approach in the R package GSVA, we analyzed immune infiltration in glioma and the correlation between infiltration level and *CENP-A* expression. To analyze the relative invasion levels of 24 immune cell types, the enrichment of published immunocyte signature genes (Bindea et al., 2013) was qualified using the expression profiling of each tumor sample. The Wilcoxon rank-sum test was employed to investigate the enrichment differences in immune cells between the *CENP-A* high and low expression groups. The association between *CENP-A* and immune cell infiltration was determined using Spearman’s correlation coefficient.

### Statistical analyses

All statistical analyses were performed using the R software (v3.6.2). Wilcoxon signed-rank and Wilcoxon rank-sum tests were performed to compare *CENP-A* expression levels in glioma and normal samples. The receiver operating characteristic (ROC) curve obtained using the pROC package was used to evaluate the effectiveness of *CENP-A* expression in discriminating between glioma and healthy samples (Robin et al., 2011). We used the Wilcoxon test, Kruskal–Wallis test, and Spearman’s correlation to evaluate the association between *CENP-A* and clinicopathological characteristics. Fisher’s exact test, Pearson’s χ^2^ test, and univariate logistic regression analyses were conducted to evaluate the correlation between *CENP-A* expression level and clinicopathological variables. Significant variables (*p* < 0.01) based on the univariate Cox regression analysis were included in the multivariate Cox regression model to identify independent prognostic parameters. Accordingly, survival curves were generated using the Kaplan–Meier method and compared using the log-rank test for each subgroup. Based on the optimal model determined by the above multivariate analysis, a nomogram was established using the R package rms to individualize the prediction of patient survival probability. The hazard ratio (HR) with a 95% confidence interval (95% CI) was used to measure the risk of individual clinical characteristics. Statistical significance was set at *p* < 0.05.

## Results

### Clinical characteristics of patients

The clinicopathological characteristics of patients with glioma collected from TCGA included age, WHO grade, isocitrate dehydrogenase (IDH) status, 1p/19q co-deletion, primary therapy outcome, gender, and histological type. A cohort of 298 females and 398 males was studied. Analysis of clinical data indicated that *CENP-A* expression was significantly associated with age (*p* < 0.001), WHO grade (*p* < 0.001), IDH status (*p* < 0.001), 1p/19q co-deletion (*p* < 0.001), primary therapy outcome (*p* < 0.001), and histological type (*p* < 0.001). No statistical association was detected between *CENP-A* expression and gender (Table 1).

**TABLE 1:** Clinical characteristics of patients with glioma from TCGA.

Characteristic	Low *CENP-A* expression	High *CENP-A* expression	*p-*value
Number of cases	348	348	
WHO grade, n (%)			<0.001
G2	188 (29.6%)	36 (5.7%)	
G3	115 (18.1%)	128 (20.2%)	
G4	6 (0.9%)	162 (25.5%)	
IDH status, n (%)			<0.001
WT	36 (5.2%)	210 (30.6%)	
Mut	309 (45%)	131 (19.1%)	
1p/19q co-deletion, n (%)			<0.001
Co-deletion	137 (19.9%)	34 (4.9%)	
No co-deletion	210 (30.5%)	308 (44.7%)	
Primary therapy outcome, n (%)			<0.001
PD	52 (11.3%)	60 (13%)	
SD	92 (19.9%)	55 (11.9%)	
PR	51 (11%)	13 (2.8%)	
CR	102 (22.1%)	37 (8%)	
Gender, n (%)			0.592
Female	153 (22%)	145 (20.8%)	
Male	195 (28%)	203 (29.2%)	
Age, n (%)			<0.001
≤60	313 (45%)	240 (34.5%)	
>60	35 (5%)	108 (15.5%)	
Histological type, n (%)			<0.001
Astrocytoma	112 (16.1%)	83 (11.9%)	
Glioblastoma	6 (0.9%)	162 (23.3%)	
Oligoastrocytoma	90 (12.9%)	44 (6.3%)	
Oligodendroglioma	140 (20.1%)	59 (8.5%)	
Age, median (IQR)	39 (32, 51)	53 (39, 63)	<0.001

WHO, world health organization; IDH, isocitrate dehydrogenase; WT, wild-type; MUT, mutated; PD, progressive disease; SD, stable disease; PR, partial response; CR, complete response.

### Centromeric protein A expression and clinical correlation in the cancer genome atlas and validation in Chinese glioma genome atlas

**FIGURE 1:**
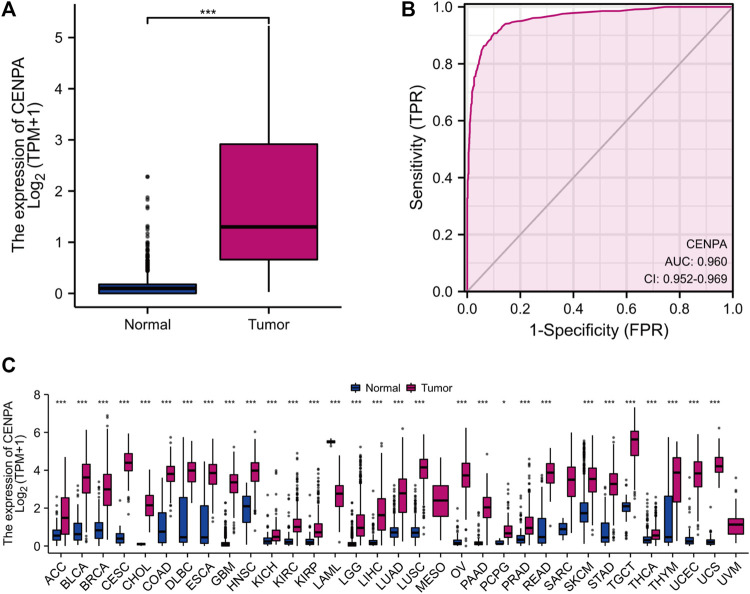
High *CENP-A* expression in tumor tissues. **(A)** *CENP-A* expression levels in glioma tissues compared with normal tissues (control). **(B)** ROC analysis of *CENP-A* expression showing its excellent ability in distinguishing tumors from normal tissues. **(C)** Pan-cancer analysis of CENP-A expression across different cancers based on TCGA data. ns, no significance, *p* > 0.05; *p < 0.05; **p < 0.01; ***p < 0.001.

**FIGURE 2:**
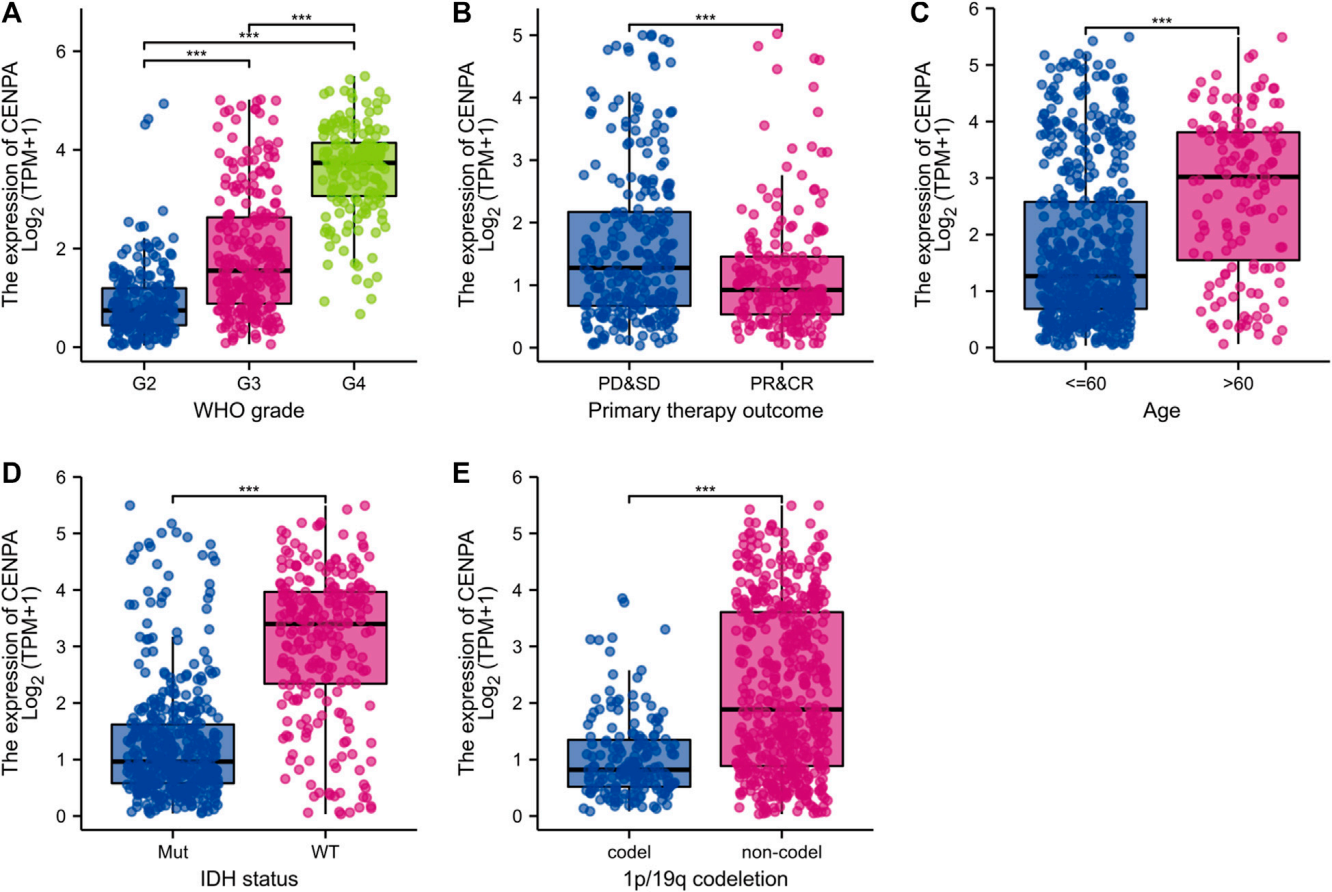
Clinical correlation analysis of *CENP-A* expression with clinicopathologic features. **(A)** WHO grade, **(B)** Primary therapy outcome, **(C)** Age, **(D)** IDH status, and **(E)** 1p/19q co-deletion. WHO, World health organization; IDH, isocitrate dehydrogenase; WT, wild type; MUT, mutated.

To compare *CENP-A* expression levels in glioma and normal samples, Wilcoxon signed-rank tests were used. *CENP-A* expression was significantly higher in glioma tissues than in healthy tissues (Figure 1A). As shown in Figure 1B, *CENP-A* expression showed excellent ability in distinguishing tumors from healthy tissues with an area under the ROC curve (AUC) of 0.960. Pan-cancer analysis consistently showed upregulated *CENP-A* expression in numerous tumor types (Figure 1C). Moreover, results based on clinical information and expression data (Figure 2) showed that, the expression level of *CENP-A* was associated with age (*p* < 0.001), WHO grade (*p* < 0.001), IDH status (*p* < 0.001), 1p/19q co-deletion (*p* < 0.001), and primary therapy outcome (*p* < 0.001). The analysis stratified by WHO grade indicated consistent results in low-grade glioma (Supplementary Figure S1). In CGGA dataset, results (Supplementary Figure S2) showed consistent association between *CENP-A* expression and WHO grade (*p* < 0.001), age (*p* < 0.001), IDH mutation (*p* < 0.001), 1p/19q co-deletion (*p* < 0.001), as well as IDH mutation & 1p/19q co-deletion status (*p* < 0.001).

To determine the correlation between *CENP-A* expression levels and clinicopathological variables, univariate logistic regression was performed. Elevated *CENP-A* expression was significantly associated with poor prognostic characteristics, including age > 60 years [OR = 4.024 (2.678–6.175) for >60 vs. ≤ 60 years], high WHO grade [OR = 49.884 (23.515–129.060) for G4 vs. G2 and G3], poor primary therapy outcome [OR = 2.444 (1.641–3.675) for progressive disease (PD) and stable disease (SD) vs. partial response (PR) and complete response (CR)], wild-type (WT) IDH [OR = 13.760 (9.247–20.963) for WT vs. mutated], and absence of 1p/19q co-deletion [OR = 5.910 (3.947–9.061) for no co-deletion vs. co-deletion], with *p* < 0.001. These results were validated using Chi-square analysis (Table 2). Our observations suggest that gliomas with upregulated *CENP-A* expression are prone to poor clinicopathological factors and a high degree of malignancy.

**TABLE 2:** Association of *CENP-A* expression with the clinicopathological characteristics of patients with glioma (logistic regression).

Characteristics	Total (n)	Odds ratio (OR)	*p*-value
WHO grade (G4 vs. G2 and G3)	635	49.884 (23.515–129.060)	<0.001
Primary therapy outcome (PD and SD vs. PR and CR)	462	2.444 (1.641–3.675)	<0.001
IDH status (WT vs. Mut)	686	13.760 (9.247–20.963)	<0.001
1p/19q co-deletion (no co-deletion vs. co-deletion)	689	5.910 (3.947–9.061)	<0.001
Age (>60 vs. ≤60)	696	4.024 (2.678–6.175)	<0.001
Gender (Male vs. Female)	696	1.098 (0.813–1.484)	0.540

WHO, world health organization; IDH, isocitrate dehydrogenase; WT, wild-type; MUT, mutated; PD, progressive disease; SD, stable disease; PR, partial response; CR, complete response.

### Centromeric protein A was an independent prognostic factor for glioma patients

**FIGURE 3:**
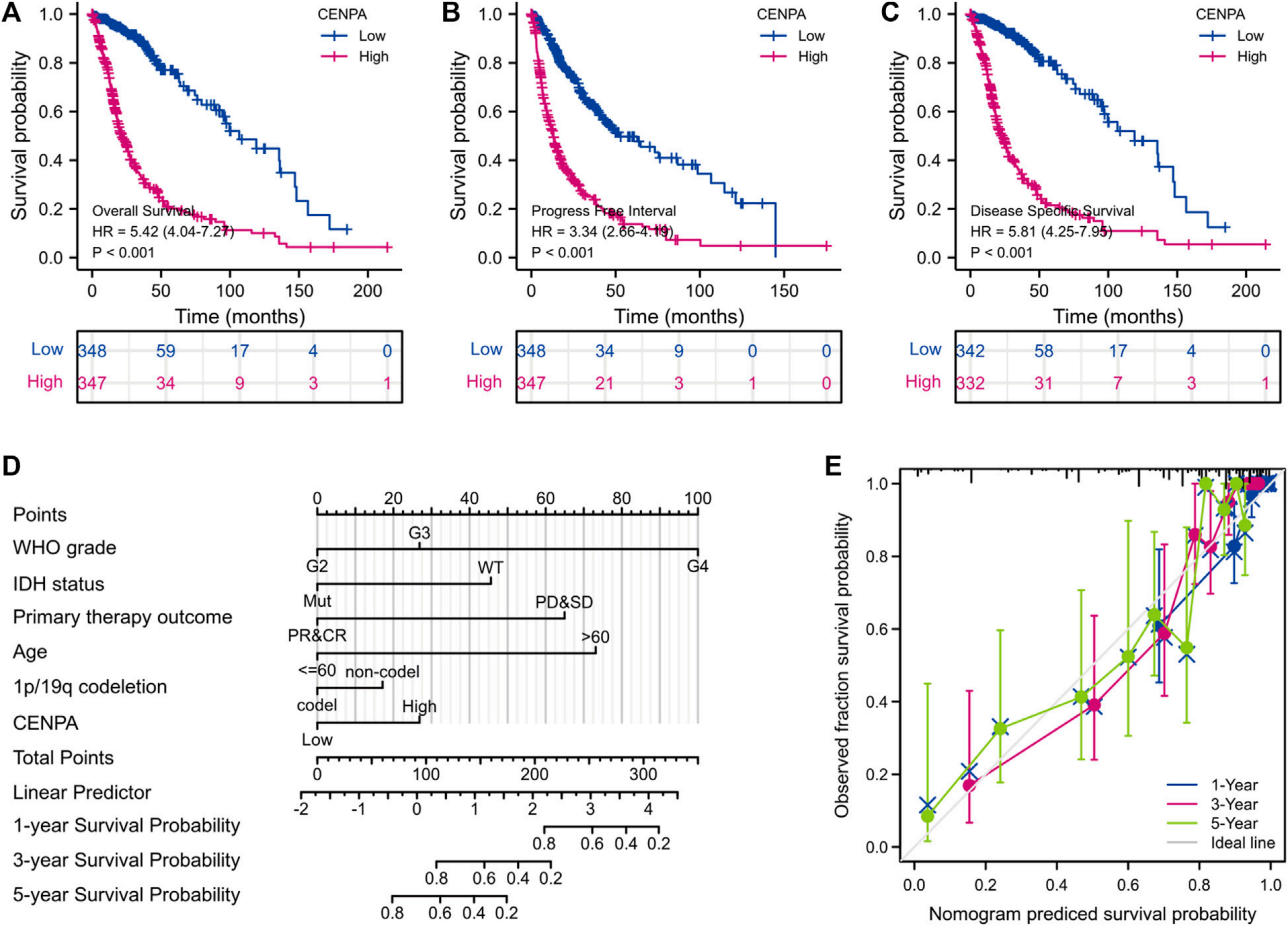
Survival analyses and prognostic nomogram. **(A–C)** Impact of *CENP-A* on the overall, progression-free, and disease-specific survival rates in glioma according to TCGA. **(D,E)** Development and verification of a glioma predictive nomogram based on *CENP-A* expression levels and independent prognostic factors.

To determine the correlation between *CENP-A* expression and survival of patients with gliomas, univariate and multivariate analyses were performed. As shown in Figures 3A–C, Kaplan–Meier survival analysis indicated that glioma cases with elevated *CENP-A* expression had a worse prognosis than those with low *CENP-A* levels (*p* < 0.001). Univariate assessment revealed that high *CENP-A* expression was markedly correlated with shorter OS (HR: 5.42; 95% CI: 4.04–7.27; *p* < 0.001), poor disease-specific survival (HR: 5.81; 95% CI: 4.25–7.95; *p* < 0.001), and poor progression-free interval (HR: 3.34; 95% CI: 2.66–4.19; *p* < 0.001). Additionally, multivariate analysis supported an independent correlation between *CENP-A* and OS (HR: 1.967; 95% CI: 1.280–3.025; *p* < 0.002) as well as between OS and age, WHO grade, primary therapy outcome, and IDH status (Table 3). Therefore, elevated *CENP-A* expression was of prognostic significance in glioma.

**TABLE 3:** The correlation of *CENP-A* and clinicopathologic characteristics with overall survival in patients with glioma in TCGA, and the multivariate survival model based on univariate selection (Cox regression).

Characteristics	Total (n)	Univariate analysis		Multivariate analysis	
		Hazard ratio (95% CI)	*p*-value	Hazard ratio (95% CI)	*p*-value
WHO grade	634				
G2 and G3	466	Reference			
G4	168	9.496 (7.212–12.503)	<0.001	4.106 (1.429–11.794)	0.009
IDH status	685				
Mut	439	Reference			
WT	246	8.551 (6.558–11.150)	<0.001	2.708 (1.712–4.282)	<0.001
1p/19q co-deletion	688				
No co-deletion	518	Reference			
Co-deletion	170	0.226 (0.147–0.347)	<0.001	0.736 (0.422–1.285)	0.281
Primary therapy outcome	461				
PD&SD	259	Reference			
PR&CR	202	0.209 (0.120–0.366)	<0.001	0.302 (0.164–0.559)	<0.001
Age	695				
≤60	552	Reference			
>60	143	4.668 (3.598–6.056)	<0.001	4.116 (2.548–6.647)	<0.001
CENP-A	695				
Low	348	Reference			
High	347	5.422 (4.044–7.271)	<0.001	1.967 (1.280–3.025)	0.002

WHO, world health organization; IDH, isocitrate dehydrogenase; WT, wild-type; MUT, mutated; PD, progressive disease; SD, stable disease; PR, partial response; CR, complete response.

### Development and validation of a centromeric protein A based prognostic prediction nomogram

To predict the survival of glioma individuals using a visualized approach, a nomogram was created by integrating *CENP-A* expression and other independent prognostic factors including age, WHO grade, primary therapy outcome, IDH status, and 1p/19q co-deletion (Figure 3D), which were determined by the above multivariate Cox analysis. A lower survival probability was represented by a higher value of total points accumulated from the points of all variables on the nomogram. A calibration plot for survival probabilities showed that nomogram prediction well agrees with observed fraction (Figure 3E). Our prognostic nomogram achieved promising predicting efficacy for the 1-, 3-, and 5-years survival probabilities. Moreover, Harrell’s concordance index (C-index) for the nomogram was 0.859, with 1,000 bootstrap resamples. These findings indicate that the nomogram performs better than clinical prognostic factors in predicting the survival probability of patients with glioma.

### Effect of centromeric protein A expression on glioma prognosis in patient subgroups

**FIGURE 4:**
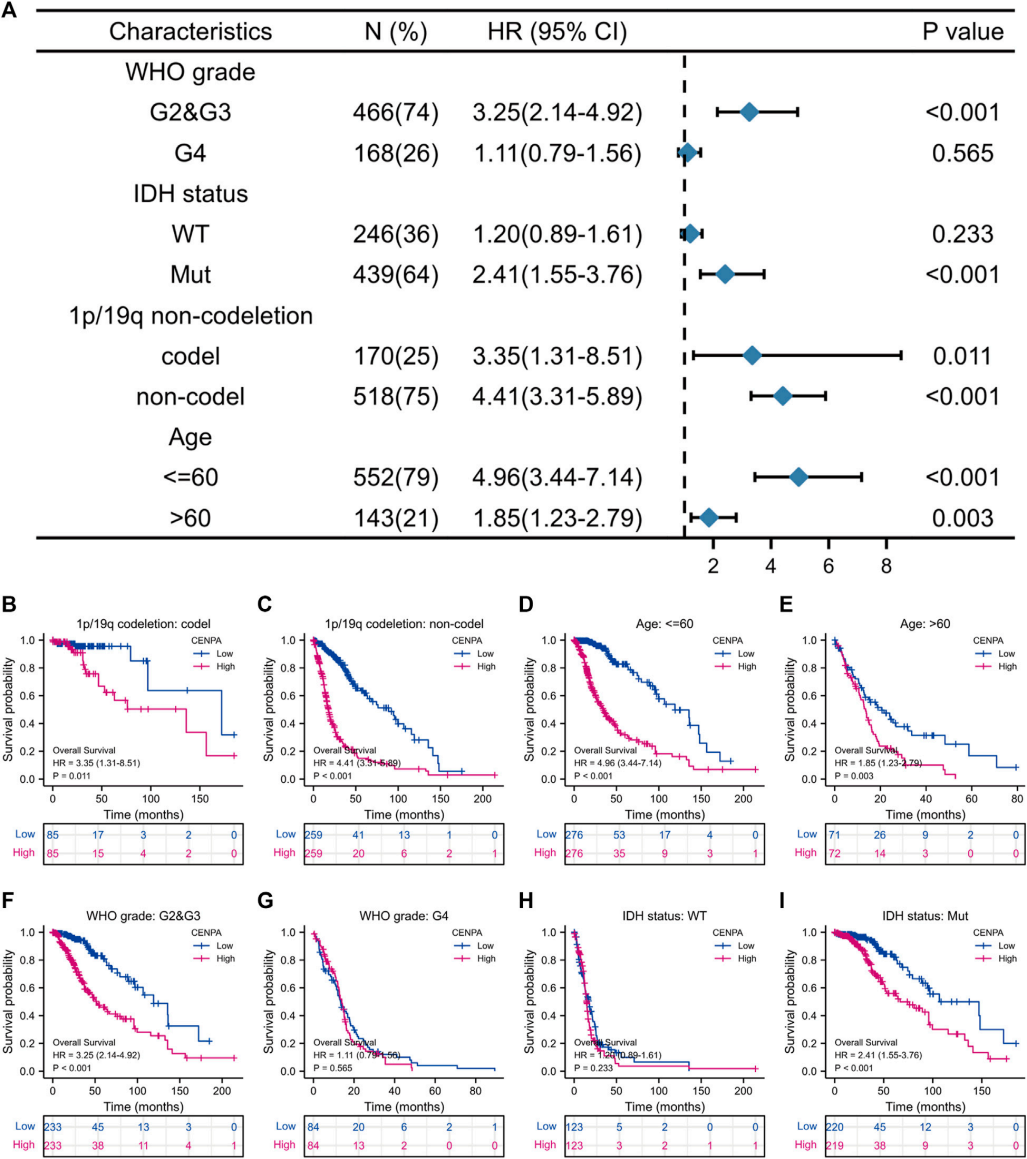
The correlation of *CENP-A* expression with glioma prognosis among patients with various clinicopathological characteristics. **(A)** Forest plots showing the subgroup analysis of overall survival. **(B–I)** Kaplan–Meier survival curves of each patient subgroup.

To better assess the prognostic ability of *CENP-A*, the relationship between *CENP-A* expression and patient survival in subgroups stratified by clinicopathological characteristics was investigated using univariate Cox analysis (Figure 4). High *CENP-A* expression was associated with shorter OS among patients with different 1p/19q co-deletion statuses and age (Figures 4B–E). Additionally, elevated *CENP-A* expression was specifically associated with decreased OS in patients with mutated IDH [HR = 2.41 (1.55–3.76), *p* < 0.001] and in patients with WHO grades G2 and G3 [HR = 3.25 (2.14–4.92), *p* < 0.001], but not in other subgroups (Figures 4F–I). Glioma patients in CGGA showed similar results, especially within WHO grade G2 and G3 cohort (*p* < 0.05, Supplementary Figure S3). The results confirmed that CENP-A retained its ability to predict survival among subgroups with various clinicopathological factors.

### Identification of differentially expressed genes between high- and -low centromeric protein A in patients with glioma

**FIGURE 5:**
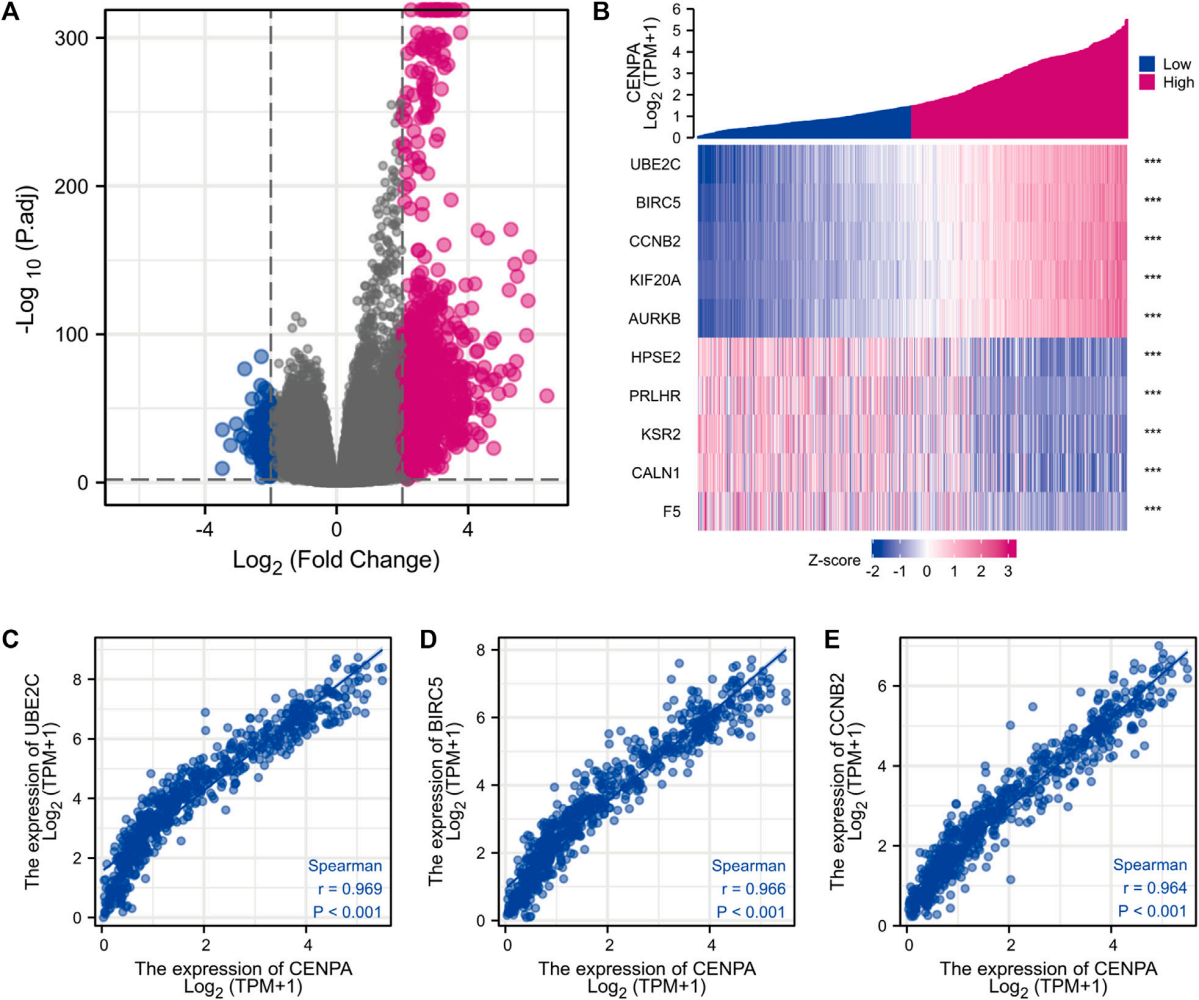
Differentially expressed genes (DEGs) between high and low *CENP-A* expression glioma groups in TCGA dataset. **(A)** Heatmap of the top five upregulated and downregulated DEGs. **(B)** Volcano plot of DEGs expression profiles. **(C–E)** Scatter plot showing the correlation between *CENP-A* expression and *UBE2C* **(C),** *BIRC5* **(D),** and *CCNB2* **(E)** expression.

Based on threshold values (|log2 FC| > 2 and adjusted *p* < 0.01) (Love et al., 2014), DEGs between high- and -low *CENP-A* were identified after an analysis of HTSeq-Counts data from TCGA using the R package DESeq2. DEGs are presented in a heatmap and volcano plot (Figure 5). A total of 521 DEGs (460 upregulated and 61 downregulated) that correlated with *CENP-A* are included in the volcano plot (Figure 5A). The top and bottom five genes in the heatmap showed significantly positive and negative correlations with *CENP-A* expression, respectively (Figure 5B). Among the DEGs, *CENP-A* was positively correlated with UBE2C (Spearman’s *r* = 0.969, *p* < 0.001), BIRC5 (Spearman’s *r* = 0.966, *p* < 0.001), and CCNB2 (Spearman’s *r* = 0.964, *p* < 0.001) (Figures 5C–E). UBE2C, BIRC5, and CCNB2 were reported to be oncogenic and are associated with several cancers including glioma (Renner et al., 2016; Dastsooz et al., 2019; Wang et al., 2021b). These results suggest the involvement of *CENP-A* in a wide array of pathways and processes through gene regulation.

### Gene ontology and kyoto encyclopedia of genes and genomes functional enrichment and protein-protein interaction network analyses of differentially expressed genes

**FIGURE 6:**
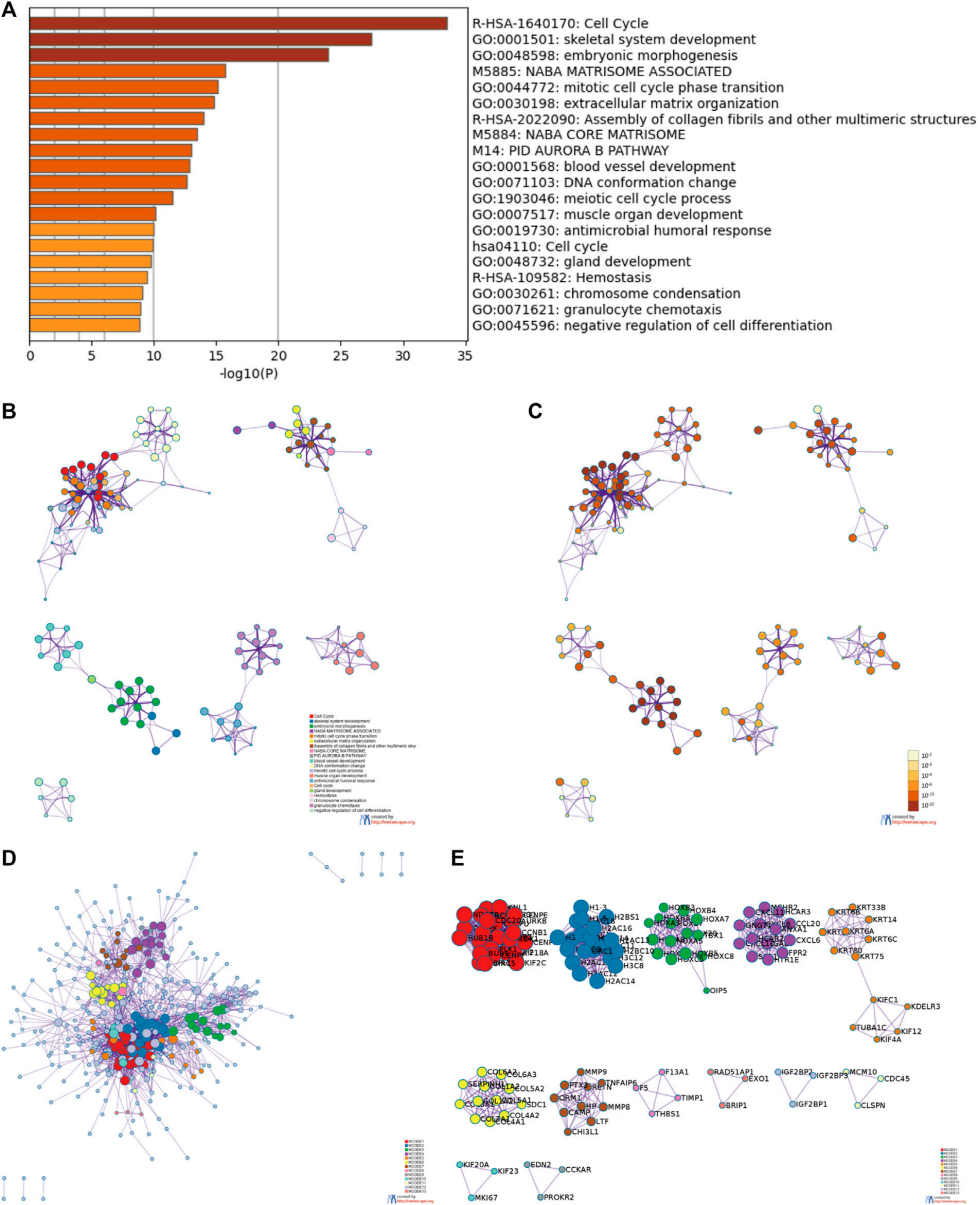
Functional enrichment and protein-protein interaction (PPI) enrichment analyses of *CENP-A*-related DEGs. **(A)** Heatmap showing Gene Ontology (GO) functional enrichment analysis. **(B,C)** Visualized network of top 20 GO enriched terms. **(D,E)** PPI networks and the most significant Molecular Complex Detection (MCODE) sub-networks.

For an in-depth understanding of the identified DEGs, we proceeded to GO and KEGG functional enrichment analyses using Metascape tools and found that 521 DEGs were involved in diverse biological processes (BP), cellular components (CC), and molecular functions. Those associated with *CENP-A-*related DEGs included cell cycle, skeletal system development, embryonic morphogenesis, Naba matrisome associated, mitotic cell cycle phase transition, extracellular matrix organization, assembly of collagen fibrils and other multimeric structures, Naba core matrisome, and PID aurora b pathway (Figure 6). Accordingly, *CENP-A-*specific DEGs were closely associated with cell cycle progression. Furthermore, PPI networks were constructed in Metascape to identify protein interactions between DEGs and better illuminate the biological significance (Figure 6D). To derive more biologically interpretable results, the most significant MCODE sub-networks that are highly interlinked were extracted from PPI, and each complex was assigned a unique color (Figure 6E). These pathways or processes may provide clues for exploring the potential functions of *CENP-A* in glioma.

### Identification of centromeric protein A-related signaling pathways

**FIGURE 7:**
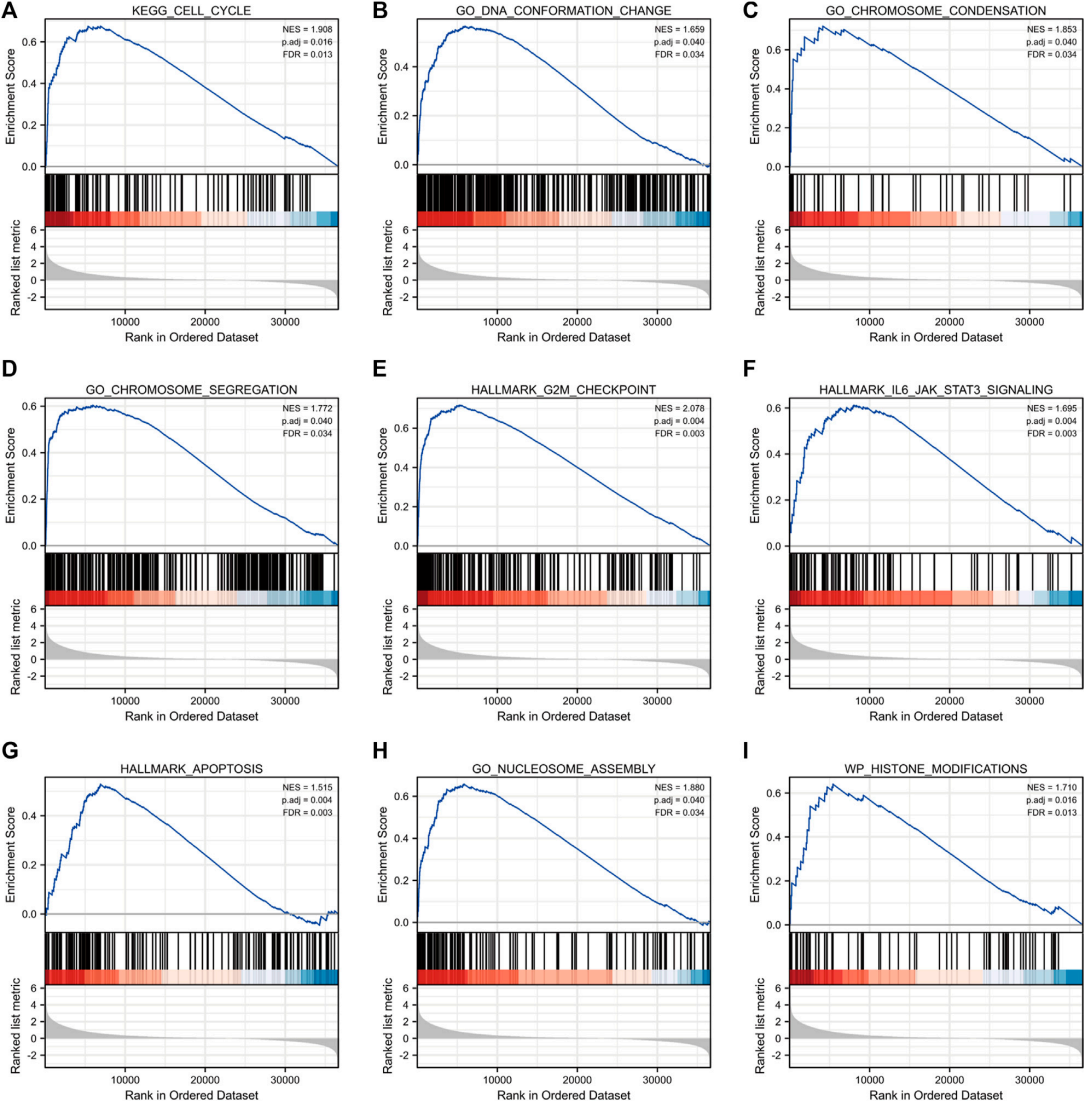
Gene set enrichment analysis (GSEA) enrichment plots including **(A)** cell cycle, **(B)** DNA conformation change, **(C)** chromosome condensation, **(D)** chromosome segregation, **(E)** G2M checkpoint, **(F)** IL6-JAK-STAT3 signaling, **(G)** apoptosis, **(H)** nucleosome assembly and **(I)** histone modifications.

GSEA of high and low *CENP-A* expression datasets was conducted to identify the critical signaling pathways or phenotypes involved in gliomas. There was a significant differential enrichment of numerous pathways within the MSigDB collection (c2.cp.v7.2.symbols, h. all.v7.2.symbols, and c5.all.v7.2.symbols) with a threshold of FDR < 0.25 and adjusted *p* < 0.05. As shown in Figure 7, the signaling cascades, including cell cycle, DNA conformation change, chromosome condensation, chromosome segregation, G2M checkpoint, IL6-JAK-STAT3 signaling, apoptosis, nucleosome assembly, and histone modifications, were enriched in the high-*CENP-A* group, thereby highlighting the potential functions of *CENP-A* in gliomagenesis.

### Correlation of centromeric protein A with immune infiltration

**FIGURE 8:**
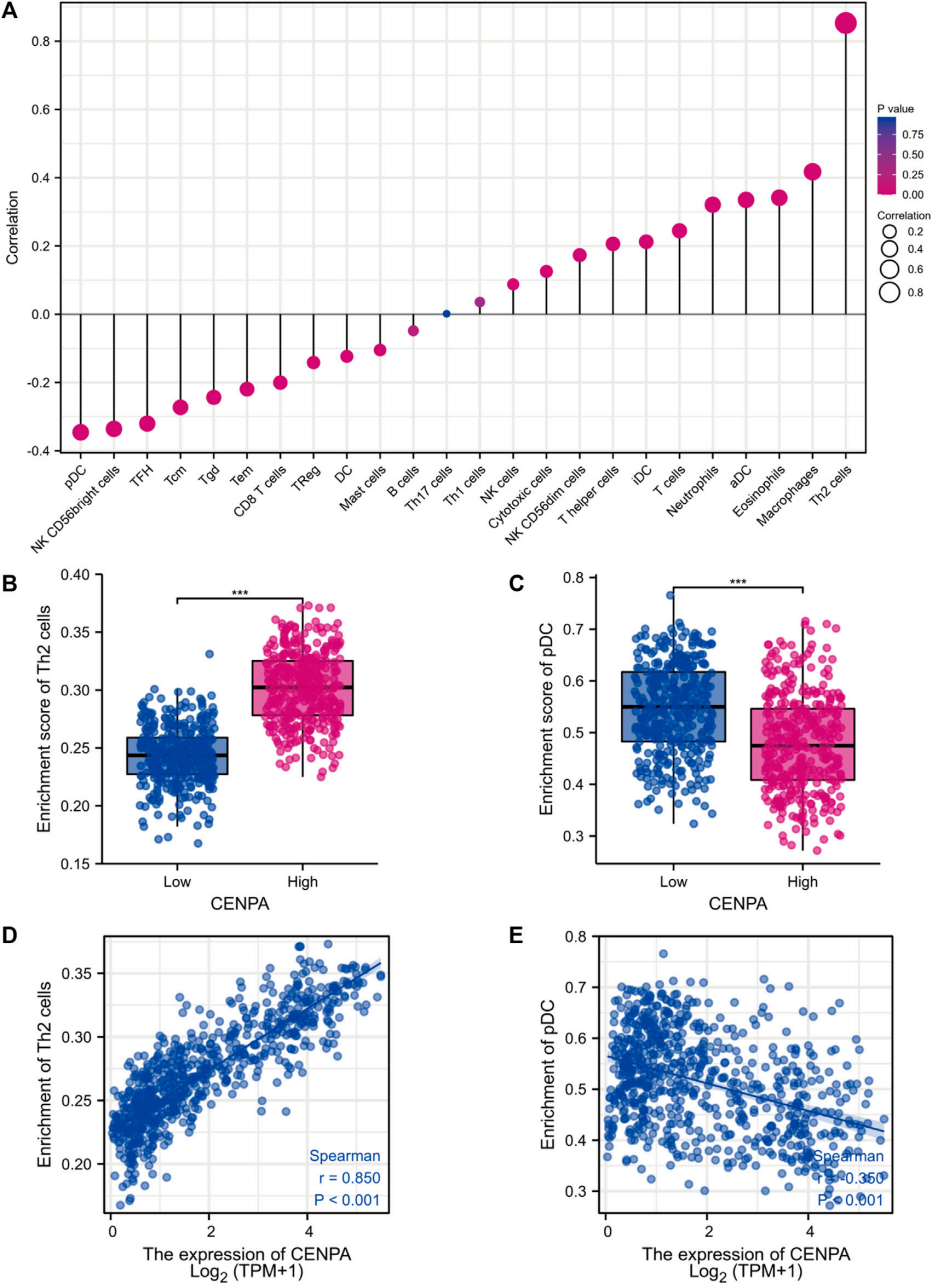
The role of CENP-A in tumor immune responses. **(A)** A forest plot showing the association between *CENP-A* expression and immune infiltration level. **(B,C)** The abundances of Th2 cells and pDC cells among low- and high-*CENP-A* expression groups. **(D,E)** The correlation between *CENP-A* expression levels and the relative enrichment levels of Th2 and pDC cells.

Brain tumor immunity has gained increased attention for its vital role in affecting therapeutic response and prognosis (Sampson et al., 2020). We further explored the correlation between *CENP-A* expression and immunocyte enrichment levels quantified by ssGSEA in the glioma tumor microenvironment *via* Spearman correlation. The results showed that Th2 cells had a remarkable positive correlation with *CENP-A* expression (Spearman’s *r* = 0.85, *p* < 0.001; Figure 8). Moreover, as illustrated by the Wilcoxon rank-sum test, the enrichment score of Th2 cells was significantly higher in high-*CENP-A* samples than in low-CENP-A samples. The relative abundance of other immune cell populations, including plasmacytoid dendritic cells (pDCs), macrophages, eosinophils, and activated dendritic cells (aDCs), was moderately correlated with *CENP-A* expression. Glioma data from CGGA showed similar correlation trend between *CENP-A* expression and infiltration of Th2 cells and pDCs (Supplementary Figure S4).

## Discussion

At present, the study of gene expression profiles in glioma has been widely applied to explore glioma pathogenesis and management. Identifying molecular markers is a promising way to guide decision-making and improve prognosis for clinical glioma. *CENP-A* regulates centromere protein assembly and is essential for progression through chromosome segregation, mitosis, and cell division. Experiments have demonstrated that excess *CENP-A* accumulates ectopically in the human cancer genome (Athwal et al., 2015). Accumulating evidences have verified that the overexpression of *CENP-A* becomes a common result in a growing body of research on cancers (Renaud-Pageot et al., 2022). *CENP-A* overexpression plays a pivotal role in chromosomal instability and pathogenesis of malignancies through chromosome segregation defects (Shrestha et al., 2017), a mechanism involved *CENP-A* in cancers (Sun et al., 2016; Sharma et al., 2019). In addition, elevated *CENP-A* levels promote the proliferation of cancer cells in hepatoma (Li et al., 2011), prostate (Saha et al., 2020) and renal cell carcinoma (Wang et al., 2021a). The observations support an association of *CENP-A* function with cell proliferation. Besides a role in cell proliferation following malignant transformation, in the cellular context of defective p53 (Filipescu et al., 2017; Jeffery et al., 2021), *CENP-A* overexpression stimulates epithelial-mesenchymal transition, a major contributing factor in the metastasis of cancer cells (Jeffery et al., 2021). *CENP-A* expression was positively associated with cancer metastasis in gastric (Xu et al., 2020) and renal (Wang et al., 2021a) cancers. Taking the evidence together, we believe that *CENP-A* overexpression is related to malignant transformation, tumor invasiveness and metastasis in specific cancer contexts. In reports with patient data, elevated *CENP-A* levels prognosticate poor patient survival for cancers (Zhang et al., 2016; Saha et al., 2020) and patient outcome after chemotherapy (Zhang et al., 2016). However, the clinical significance of *CENP-A* in glioma, especially its expression pattern and prognostic role, has not yet been systematically explored. In our study, bioinformatic analyses of TCGA RNA-sequencing data combining GBM and low-grade glioma confirmed increased *CENP-A* expression which was associated with malignant clinicopathological status (high WHO grade, primary therapy outcome of PD&SD, age > 60, WT IDH status, and absence of 1p/19q co-deletion), short survival time, and poor prognosis. The results were further validated in CGGA dataset. Furthermore, GSEA revealed that the high-*CENP-A* phenotype showed differential enrichment of pathways including cell cycle, DNA conformation change, chromosome condensation, chromosome segregation, G2M checkpoint, IL6-JAK-STAT3 signaling, apoptosis, nucleosome assembly, and histone modifications. Immune filtration analysis suggested that the expression of *CENP-A* correlated with immune infiltration status. Our results support *CENP-A* as a potential prognostic biomarker for gliomas.

In subgroup analysis, *CENP-A* expression remained correlated with poor prognosis in subsets grouped by WHO grade, IDH, 1p/19q co-deletion, and age statuses, which strongly suggests that *CENP-A* is a glioma grading biomarker within these subsets. We found a marked association between expression levels of *CENP-A* and OS in all 1p/19q co-deletion and age subgroups. Notably, there was a significant association between *CENP-A* expression levels and OS in grades 2 and 3 and mutated IDH status, but not in grade 4 and wild-type IDH status. The results from CGGA dataset validated this association in glioma, as well as grade 2 and 3 subgroups. These findings indicate that the association between *CENP-A* expression levels and survival varies by WHO grade and IDH status, and high *CENP-A* expression is more likely to negatively impact the survival of patients with low-grade gliomas.

The Cox model showed that *CENP-A* was an independent prognostic predictor of glioma. Subsequently, a nomogram was developed for the accurate prediction of prognosis with a personalized score for individual patients, and the model combined *CENP-A* with other predictors, including WHO grade, primary therapy outcome, age, IDH status, and 1p/19q co-deletion. Glioma with high WHO grade, IDH-wild-type, 1p/19q-non-codeleted and primary therapy outcome of PD are inclined to adverse survival (Eckel-Passow et al., 2015; Weller et al., 2015). IDH and 1p/19q co-deletion statuses were determined as classifying factors in the 2016 WHO diagnostic criteria for gliomas. IDH-mutant and 1p/19q-codeleted have been regarded as clinically relevant biomarkers in lower-grade gliomas with a favorable prognosis (Brat et al., 2015). 1p/19q co-deletion status is especially associated with patient outcomes in response to adjuvant chemotherapy (van den Bent et al., 2013). Age at diagnosis affects incidence rates of glioma remarkably. Older Age is associated with worse glioma survival and the effect on survival differs in glioma subtypes (Ostrom et al., 2019). The C-index and calibration plot confirmed that the nomogram performed well in predicting the 1-, 3-, and 5-year survival of patients with glioma. Therefore, our nomogram is a valuable clinical prognostic model.

To further investigate *CENP-A* function, GSEA was performed which showed that *CENP-A* was associated with cell cycle regulation, chromosome segregation, and nucleosome assembly in glioma. Previous studies have revealed that defects in chromosome segregation can lead to the phenotypes observed in tumor cells. Additionally, chromosomal instability induced by abnormal nucleosome assembly and chromosome segregation in the cell cycle may contribute to the progression of glioma (Milinkovic et al., 2012; Ferguson et al., 2015). Our results revealed an association between *CENP-A* and apoptosis, which is consistent with a previous study showing that nucleosome assembly failure is correlated with radiation-induced GBM cell death (Serafim et al., 2020). Moreover, HJURP is recognized as a *CENP-A*-specific chaperone, and its overexpression often accompanies the overexpression of *CENP-A* (Foltz et al., 2009). HJURP knockdown increases radiation-induced apoptosis in glioblastoma cells (Serafim et al., 2020). The functional enrichment analysis with Metascape (Zhou et al., 2019) showed consistent results that found an enrichment in cell cycle. Therefore, *CENP-A* may play a role in the cell cycle regulation to promote the survival and proliferation of glioma cells. However, it remains unclear whether elevated *CENP-A* levels contribute to glioma progression by inducing chromosomal instability.

Additionally, we revealed that high *CENP-A* expression phenotype was strongly associated with the inflammation-related IL6-JAK-STAT3 signaling pathway, which is associated with poor prognosis in patients with glioma (Yao et al., 2016). In the tumor immune microenvironment, the IL6-JAK-STAT3 pathway is hyperactivated in a multitude of cancers, which suppresses the anti-tumor immune response and promotes tumor progression (Yao et al., 2016). Preclinical and clinical investigations showed that IL6-JAK-STAT3 pathway inhibition has therapeutic benefits in cancer and that STAT3 inhibition inhibits the growth of glioma cells (Shen et al., 2009; Johnson et al., 2018). Nevertheless, the regulatory mechanisms underlying these associated functions and pathways remain poorly characterized and require further research.

Tumor immunosuppressive microenvironment represents an important factor of cancer progression and poor prognosis in glioma (Jackson et al., 2019). Since the prognostic role of infiltrating immune cells have been proposed across many human cancers (Gentles et al., 2015), we pay attention to the link between glioma immunity and *CENP-A* in this research. Several research models regarding centromeric factor in cancer proposed the association with immune infiltration (Shi et al., 2021; Zeng et al., 2021; Zhou et al., 2021). We also showed that *CENP-A* expression level was associated with the level of infiltrating immune cells in gliomas and presented the strongest correlation with Th2 cells and pDCs. Tumor-specific Th2 cell responses are associated with tumor immune evasion, and Th2 cytokines such as IL-4 and IL-13 are implicated in the suppression of host immune effector responses to tumors (Gordon and Martinez, 2010; Tosolini et al., 2011). Th2 cytokines are strongly expressed in glioma cell lines and GBM samples (Hao et al., 2002). Moreover, a strong Th2-bias response was reported in patients with gliomas, especially those with recurrent GBM(Shimato et al., 2012; Harshyne et al., 2016), and the Th2 phenotype is associated with a poor prognosis in patients with glioma (Piperi et al., 2011; Shimato et al., 2012). This is consistent with our results that found an increased enrichment of Th2 cell in high *CENP-A* expression, which predicted poor prognosis. Additionally, pDCs play a suppressive role in tumors and impaired pDC activity is implicated in reduced immune responses in tumors (Reizis, 2019). Diffuse low-grade glioma with a better outcome showed elevated pDC level (Wu et al., 2020). pDCs induce anti-tumor therapeutic efficacy in GBM by producing IFN-α (Candolfi et al., 2012). Therefore, elevated *CENP-A* expression may induce Th2 cell infiltration and pDC deficiency in the tumor microenvironment, which contributes to immunotherapy resistance and poor treatment response in glioma. Collectively, our results indicate the potential role of *CENP-A* in modulating glioma-related immune responses; however, the underlying regulatory mechanisms require further investigation.

The present study has several limitations. First, it was based on open tumor databases and bioinformatics analysis and was not validated *in vitro* or *in vivo*. Second, the analysis was conducted only on the expression profiles at the mRNA level, not protein expression levels. Therefore, our results need to be validated using CENP-A protein expression levels and subsequent laboratory experiments.

In summary, our findings highlight the prognostic value and immune relevance of *CENP-A* in glioma, supporting its exploration as a potential biomarker for prognosis or a target for molecular targeted therapy. Furthermore, *CENP-A* may contribute to glioma progression through the regulation of pathways, including the cell cycle, nucleosome assembly, IL6-JAK-STAT3 signaling, and DNA repair. Further studies are required to elucidate the clinicopathological and biological significance of *CENP-A* expression. This study provides new insights into the molecular pathogenesis and individualized treatment of gliomas.

## Data Availability

The datasets presented in this study can be found in online repositories. The names of the repository/repositories and accession number(s) can be found in the article/Supplementary Material.

## References

[B1] Amato A. Schillaci T. Lentini L. Di Leonardo A. (2009). CENPA overexpression promotes genome instability in pRb-depleted human cells. Mol. Cancer 8, 119. 10.1186/1476-4598-8-119 20003272 PMC2797498

[B2] Athwal R. K. Walkiewicz M. P. Baek S. Fu S. Bui M. Camps J. (2015). CENP-A nucleosomes localize to transcription factor hotspots and subtelomeric sites in human cancer cells. Epigenetics Chromatin 8, 2. 10.1186/1756-8935-8-2 25788983 PMC4363203

[B3] Bi J. Chowdhry S. Wu S. Zhang W. Masui K. Mischel P. S. (2020). Altered cellular metabolism in gliomas - an emerging landscape of actionable co-dependency targets. Nat. Rev. Cancer 20 (1), 57–70. 10.1038/s41568-019-0226-5 31806884

[B4] Bindea G. Mlecnik B. Tosolini M. Kirilovsky A. Waldner M. Obenauf A. C. (2013). Spatiotemporal dynamics of intratumoral immune cells reveal the immune landscape in human cancer. Immunity 39 (4), 782–795. 10.1016/j.immuni.2013.10.003 24138885

[B5] Brat D. J. Verhaak R. G. Aldape K. D. Yung W. K. Salama S. R. Cooper L. A. (2015). Comprehensive, integrative genomic analysis of diffuse lower-grade gliomas. N. Engl. J. Med. 372 (26), 2481–2498. 10.1056/NEJMoa1402121 26061751 PMC4530011

[B6] Candolfi M. King G. D. Yagiz K. Curtin J. F. Mineharu Y. Muhammad A. K. (2012). Plasmacytoid dendritic cells in the tumor microenvironment: Immune targets for glioma therapeutics. Neoplasia 14 (8), 757–770. 10.1593/neo.12794 22952428 PMC3431182

[B7] Chen X. Pan Y. Yan M. Bao G. Sun X. (2020). Identification of potential crucial genes and molecular mechanisms in glioblastoma multiforme by bioinformatics analysis. Mol. Med. Rep. 22 (2), 859–869. 10.3892/mmr.2020.11160 32467990 PMC7339479

[B8] Dastsooz H. Cereda M. Donna D. Oliviero S. (2019). A comprehensive bioinformatics analysis of UBE2C in cancers. Int. J. Mol. Sci. 20 (9), E2228. 10.3390/ijms20092228 31067633 PMC6539744

[B9] Eckel-Passow J. E. Lachance D. H. Molinaro A. M. Walsh K. M. Decker P. A. Sicotte H. (2015). Glioma groups based on 1p/19q, IDH, and TERT promoter mutations in tumors. N. Engl. J. Med. 372 (26), 2499–2508. 10.1056/NEJMoa1407279 26061753 PMC4489704

[B10] Ferguson L. R. Chen H. Collins A. R. Connell M. Damia G. Dasgupta S. (2015). Genomic instability in human cancer: Molecular insights and opportunities for therapeutic attack and prevention through diet and nutrition. Semin. Cancer Biol. 35, S5-S24–S24. 10.1016/j.semcancer.2015.03.005 25869442 PMC4600419

[B11] Filipescu D. Naughtin M. Podsypanina K. Lejour V. Wilson L. Gurard-Levin Z. A. (2017). Essential role for centromeric factors following p53 loss and oncogenic transformation. Genes. Dev. 31 (5), 463–480. 10.1101/gad.290924.116 28356341 PMC5393061

[B12] Foltz D. R. Jansen L. E. Bailey A. O. Yates J. R. 3rd Bassett E. A. Wood S. (2009). Centromere-specific assembly of CENP-a nucleosomes is mediated by HJURP. Cell 137 (3), 472–484. 10.1016/j.cell.2009.02.039 19410544 PMC2747366

[B13] Gentles A. J. Newman A. M. Liu C. L. Bratman S. V. Feng W. Kim D. (2015). The prognostic landscape of genes and infiltrating immune cells across human cancers. Nat. Med. 21 (8), 938–945. 10.1038/nm.3909 26193342 PMC4852857

[B14] Gordon S. Martinez F. O. (2010). Alternative activation of macrophages: Mechanism and functions. Immunity 32 (5), 593–604. 10.1016/j.immuni.2010.05.007 20510870

[B15] Hao C. Parney I. F. Roa W. H. Turner J. Petruk K. C. Ramsay D. A. (2002). Cytokine and cytokine receptor mRNA expression in human glioblastomas: Evidence of Th1, Th2 and Th3 cytokine dysregulation. Acta Neuropathol. 103 (2), 171–178. 10.1007/s004010100448 11810184

[B16] Harshyne L. A. Nasca B. J. Kenyon L. C. Andrews D. W. Hooper D. C. (2016). Serum exosomes and cytokines promote a T-helper cell type 2 environment in the peripheral blood of glioblastoma patients. Neuro. Oncol. 18 (2), 206–215. 10.1093/neuonc/nov107 26180083 PMC4724173

[B17] Hoffmann S. Izquierdo H. M. Gamba R. Chardon F. Dumont M. Keizer V. (2020). A genetic memory initiates the epigenetic loop necessary to preserve centromere position. EMBO J. 39 (20), e105505. 10.15252/embj.2020105505 32945564 PMC7560200

[B18] Jackson C. M. Choi J. Lim M. (2019). Mechanisms of immunotherapy resistance: lessons from glioblastoma. Nat. Immunol. 20 (9), 1100–1109. 10.1038/s41590-019-0433-y 31358997

[B19] Jeffery D. Gatto A. Podsypanina K. Renaud-Pageot C. Ponce Landete R. Bonneville L. (2021). CENP-A overexpression promotes distinct fates in human cells, depending on p53 status. Commun. Biol. 4 (1), 417. 10.1038/s42003-021-01941-5 33772115 PMC7997993

[B20] Johnson D. E. O'Keefe R. A. Grandis J. R. (2018). Targeting the IL-6/JAK/STAT3 signalling axis in cancer. Nat. Rev. Clin. Oncol. 15 (4), 234–248. 10.1038/nrclinonc.2018.8 29405201 PMC5858971

[B21] Lacoste N. Woolfe A. Tachiwana H. Garea A. V. Barth T. Cantaloube S. (2014). Mislocalization of the centromeric histone variant CenH3/CENP-A in human cells depends on the chaperone DAXX. Mol. Cell 53 (4), 631–644. 10.1016/j.molcel.2014.01.018 24530302

[B22] Li B. Pu K. Wu X. (2019). Identifying novel biomarkers in hepatocellular carcinoma by weighted gene co-expression network analysis. J. Cell. Biochem. 120, 11418–11431. 10.1002/jcb.28420 30746803

[B23] Li Y. Zhu Z. Zhang S. Yu D. Yu H. Liu L. (2011). ShRNA-targeted centromere protein A inhibits hepatocellular carcinoma growth. PLoS One 6 (3), e17794. 10.1371/journal.pone.0017794 21423629 PMC3058037

[B24] Liu W. T. Wang Y. Zhang J. Ye F. Huang X. H. Li B. (2018). A novel strategy of integrated microarray analysis identifies CENPA, CDK1 and CDC20 as a cluster of diagnostic biomarkers in lung adenocarcinoma. Cancer Lett. 425, 43–53. 10.1016/j.canlet.2018.03.043 29608985

[B25] Love M. I. Huber W. Anders S. (2014). Moderated estimation of fold change and dispersion for RNA-seq data with DESeq2. Genome Biol. 15 (12), 550. 10.1186/s13059-014-0550-8 25516281 PMC4302049

[B26] Milinkovic V. Bankovic J. Rakic M. Milosevic N. Stankovic T. Jokovic M. (2012). Genomic instability and p53 alterations in patients with malignant glioma. Exp. Mol. Pathol. 93 (2), 200–206. 10.1016/j.yexmp.2012.05.010 22664273

[B27] Ostrom Q. T. Cioffi G. Gittleman H. Patil N. Waite K. Kruchko C. (2019). CBTRUS statistical report: Primary brain and other central nervous system tumors diagnosed in the United States in 2012-2016. Neuro. Oncol. 21, v1–v100. 10.1093/neuonc/noz150 31675094 PMC6823730

[B28] Piperi C. Samaras V. Levidou G. Kavantzas N. Boviatsis E. Petraki K. (2011). Prognostic significance of IL-8-STAT-3 pathway in astrocytomas: correlation with IL-6, VEGF and microvessel morphometry. Cytokine 55 (3), 387–395. 10.1016/j.cyto.2011.05.012 21684758

[B29] Qi L. Gao C. Feng F. Zhang T. Yao Y. Wang X. (2019). MicroRNAs associated with lung squamous cell carcinoma: New prognostic biomarkers and therapeutic targets. J. Cell. Biochem. 120 (11), 18956–18966. 10.1002/jcb.29216 31241205

[B30] Quevedo R. Spreafico A. Bruce J. Danesh A. El Ghamrasni S. Giesler A. (2020). Centromeric cohesion failure invokes a conserved choreography of chromosomal mis-segregations in pancreatic neuroendocrine tumor. Genome Med. 12 (1), 38. 10.1186/s13073-020-00730-9 32345369 PMC7189550

[B31] Rajput A. B. Hu N. Varma S. Chen C. H. Ding K. Park P. C. (2011). Immunohistochemical assessment of expression of centromere protein-A (CENPA) in human invasive breast cancer. Cancers (Basel) 3 (4), 4212–4227. 10.3390/cancers3044212 24213134 PMC3763419

[B32] Reifenberger G. Wirsching H. G. Knobbe-Thomsen C. B. Weller M. (2017). Advances in the molecular genetics of gliomas - implications for classification and therapy. Nat. Rev. Clin. Oncol. 14 (7), 434–452. 10.1038/nrclinonc.2016.204 28031556

[B33] Reizis B. (2019). Plasmacytoid dendritic cells: Development, regulation, and function. Immunity 50 (1), 37–50. 10.1016/j.immuni.2018.12.027 30650380 PMC6342491

[B34] Renaud-Pageot C. Quivy J. P. Lochhead M. Almouzni G. (2022). CENP-A regulation and cancer. Front. Cell Dev. Biol. 10, 907120. 10.3389/fcell.2022.907120 35721491 PMC9201071

[B35] Renner G. Janouskova H. Noulet F. Koenig V. Guerin E. Bär S. (2016). Integrin α5β1 and p53 convergent pathways in the control of anti-apoptotic proteins PEA-15 and survivin in high-grade glioma. Cell Death Differ. 23 (4), 640–653. 10.1038/cdd.2015.131 26470725 PMC4986636

[B36] Robin X. Turck N. Hainard A. Tiberti N. Lisacek F. Sanchez J. C. (2011). pROC: an open-source package for R and S+ to analyze and compare ROC curves. BMC Bioinforma. 12, 77. 10.1186/1471-2105-12-77 PMC3068975 21414208

[B37] Saha A. K. Contreras-Galindo R. Niknafs Y. S. Iyer M. Qin T. Padmanabhan K. (2020). The role of the histone H3 variant CENPA in prostate cancer. J. Biol. Chem. 295 (25), 8537–8549. 10.1074/jbc.RA119.010080 32371391 PMC7307189

[B38] Sampson J. H. Gunn M. D. Fecci P. E. Ashley D. M. (2020). Brain immunology and immunotherapy in brain tumours. Nat. Rev. Cancer 20 (1), 12–25. 10.1038/s41568-019-0224-7 31806885 PMC7327710

[B39] Sansregret L. Vanhaesebroeck B. Swanton C. (2018). Determinants and clinical implications of chromosomal instability in cancer. Nat. Rev. Clin. Oncol. 15 (3), 139–150. 10.1038/nrclinonc.2017.198 29297505

[B40] Serafim R. B. Cardoso C. Di Cristofaro L. F. M. Pienna Soares C. Araújo Silva W. Jr. Espreafico E. M. (2020). HJURP knockdown disrupts clonogenic capacity and increases radiation-induced cell death of glioblastoma cells. Cancer Gene Ther. 27 (5), 319–329. 10.1038/s41417-019-0103-0 31138900

[B41] Sharma A. B. Dimitrov S. Hamiche A. Van Dyck E. (2019). Centromeric and ectopic assembly of CENP-A chromatin in health and cancer: Old marks and new tracks. Nucleic Acids Res. 47 (3), 1051–1069. 10.1093/nar/gky1298 30590707 PMC6379705

[B42] Shen J. Li R. Li G. (2009). Inhibitory effects of decoy-ODN targeting activated STAT3 on human glioma growth *in vivo* . Vivo 23 (2), 237–243. 19414409

[B43] Shi K. Zhu X. Wu J. Chen Y. Zhang J. Sun X. (2021). Centromere protein E as a novel biomarker and potential therapeutic target for retinoblastoma. Bioengineered 12 (1), 5950–5970. 10.1080/21655979.2021.1972080 34482803 PMC8806431

[B44] Shimato S. Maier L. M. Maier R. Bruce J. N. Anderson R. C. Anderson D. E. (2012). Profound tumor-specific Th2 bias in patients with malignant glioma. BMC Cancer 12, 561. 10.1186/1471-2407-12-561 23186108 PMC3537750

[B45] Shrestha R. L. Ahn G. S. Staples M. I. Sathyan K. M. Karpova T. S. Foltz D. R. (2017). Mislocalization of centromeric histone H3 variant CENP-A contributes to chromosomal instability (CIN) in human cells. Oncotarget 8 (29), 46781–46800. 10.18632/oncotarget.18108 28596481 PMC5564523

[B46] Shrestha R. L. Rossi A. Wangsa D. Hogan A. K. Zaldana K. S. Suva E. (2021). CENP-A overexpression promotes aneuploidy with karyotypic heterogeneity. J. Cell Biol. 220 (4), e202007195. 10.1083/jcb.202007195 33620383 PMC7905998

[B47] Stangeland B. Mughal A. A. Grieg Z. Sandberg C. J. Joel M. Nygard S. (2015). Combined expressional analysis, bioinformatics and targeted proteomics identify new potential therapeutic targets in glioblastoma stem cells. Oncotarget 6 (28), 26192–26215. 10.18632/oncotarget.4613 26295306 PMC4694895

[B48] Sun X. Clermont P. L. Jiao W. Helgason C. D. Gout P. W. Wang Y. (2016). Elevated expression of the centromere protein-A(CENP-A)-encoding gene as a prognostic and predictive biomarker in human cancers. Int. J. Cancer 139 (4), 899–907. 10.1002/ijc.30133 27062469

[B49] Tosolini M. Kirilovsky A. Mlecnik B. Fredriksen T. Mauger S. Bindea G. (2011). Clinical impact of different classes of infiltrating T cytotoxic and helper cells (Th1, th2, treg, th17) in patients with colorectal cancer. Cancer Res. 71 (4), 1263–1271. 10.1158/0008-5472.can-10-2907 21303976

[B50] van den Bent M. J. Brandes A. A. Taphoorn M. J. Kros J. M. Kouwenhoven M. C. Delattre J. Y. (2013). Adjuvant procarbazine, lomustine, and vincristine chemotherapy in newly diagnosed anaplastic oligodendroglioma: long-term follow-up of EORTC brain tumor group study 26951. J. Clin. Oncol. 31 (3), 344–350. 10.1200/jco.2012.43.2229 23071237

[B51] Vivian J. Rao A. A. Nothaft F. A. Ketchum C. Armstrong J. Novak A. (2017). Toil enables reproducible, open source, big biomedical data analyses. Nat. Biotechnol. 35 (4), 314–316. 10.1038/nbt.3772 28398314 PMC5546205

[B52] Wang Q. Xu J. Xiong Z. Xu T. Liu J. Liu Y. (2021a). CENPA promotes clear cell renal cell carcinoma progression and metastasis via Wnt/β-catenin signaling pathway. J. Transl. Med. 19 (1), 417. 10.1186/s12967-021-03087-8 34627268 PMC8502268

[B53] Wang Y. Zhang H. Wang M. He J. Guo H. Li L. (2021b). CCNB2/SASP/Cathepsin B & PGE2 Axis induce cell senescence mediated malignant transformation. Int. J. Biol. Sci. 17 (13), 3538–3553. 10.7150/ijbs.63430 34512164 PMC8416730

[B54] Weller M. Wick W. Aldape K. Brada M. Berger M. Pfister S. M. (2015). Glioma. Nat. Rev. Dis. Prim. 1, 15017. 10.1038/nrdp.2015.17 27188790

[B55] Wesseling P. Capper D. (2018). WHO 2016 Classification of gliomas. Neuropathol. Appl. Neurobiol. 44 (2), 139–150. 10.1111/nan.12432 28815663

[B56] Wu F. Wang Z. L. Wang K. Y. Li G. Z. Chai R. C. Liu Y. Q. (2020). Classification of diffuse lower-grade glioma based on immunological profiling. Mol. Oncol. 14 (9), 2081–2095. 10.1002/1878-0261.12707 32392361 PMC7463381

[B57] Wu Q. Chen Y. F. Fu J. You Q. H. Wang S. M. Huang X. (2014). Short hairpin RNA-mediated down-regulation of CENP-A attenuates the aggressive phenotype of lung adenocarcinoma cells. Cell. Oncol. 37 (6), 399–407. 10.1007/s13402-014-0199-z PMC13004451 25228009

[B58] Wu Q. Qian Y. M. Zhao X. L. Wang S. M. Feng X. J. Chen X. F. (2012). Expression and prognostic significance of centromere protein A in human lung adenocarcinoma. Lung Cancer 77 (2), 407–414. 10.1016/j.lungcan.2012.04.007 22542705

[B59] Xu Y. Liang C. Cai X. Zhang M. Yu W. Shao Q. (2020). High centromere protein-A (CENP-A) expression correlates with progression and prognosis in gastric cancer. Onco. Targets. Ther. 13, 13237–13246. 10.2147/OTT.S263512 33402833 PMC7778524

[B60] Yao Y. Ye H. Qi Z. Mo L. Yue Q. Baral A. (2016). B7-H4(B7x)-Mediated cross-talk between glioma-initiating cells and macrophages via the IL6/JAK/STAT3 pathway lead to poor prognosis in glioma patients. Clin. Cancer Res. 22 (11), 2778–2790. 10.1158/1078-0432.ccr-15-0858 27001312 PMC4891287

[B61] Yu G. Wang L. G. Han Y. He Q. Y. (2012). clusterProfiler: an R package for comparing biological themes among gene clusters. OMICS 16 (5), 284–287. 10.1089/omi.2011.0118 22455463 PMC3339379

[B62] Zeng Z. Jiang X. Pan Z. Zhou R. Lin Z. Tang Y. (2021). Highly expressed centromere protein L indicates adverse survival and associates with immune infiltration in hepatocellular carcinoma. Aging (Albany NY) 13 (19), 22802–22829. 10.18632/aging.203574 34607313 PMC8544325

[B63] Zhang W. Mao J. H. Zhu W. Jain A. K. Liu K. Brown J. B. (2016). Centromere and kinetochore gene misexpression predicts cancer patient survival and response to radiotherapy and chemotherapy. Nat. Commun. 7, 12619. 10.1038/ncomms12619 27577169 PMC5013662

[B64] Zhang W. Xu Y. Zhang J. Wu J. (2020a). Identification and analysis of novel biomarkers involved in chromophobe renal cell carcinoma by integrated bioinformatics analyses. Biomed. Res. Int. 2020, 2671281. 10.1155/2020/2671281 32090070 PMC7029304

[B65] Zhang Y. Yang L. Shi J. Lu Y. Chen X. Yang Z. (2020b). The oncogenic role of CENPA in hepatocellular carcinoma development: Evidence from bioinformatic analysis. Biomed. Res. Int. 2020, 3040839. 10.1155/2020/3040839 32337237 PMC7168693

[B66] Zhou H. Bian T. Qian L. Zhao C. Zhang W. Zheng M. (2021). Prognostic model of lung adenocarcinoma constructed by the CENPA complex genes is closely related to immune infiltration. Pathol. Res. Pract. 228, 153680. 10.1016/j.prp.2021.153680 34798483

[B67] Zhou Y. Zhou B. Pache L. Chang M. Khodabakhshi A. H. Tanaseichuk O. (2019). Metascape provides a biologist-oriented resource for the analysis of systems-level datasets. Nat. Commun. 10 (1), 1523. 10.1038/s41467-019-09234-6 30944313 PMC6447622

