# Supplementary material for: CENP-A is a potential prognostic biomarker and correlated with immune infiltration levels in glioma patients
Source: Front Genet. 2022 Aug 29;13:931222. doi: 10.3389/fgene.2022.931222 (PMC9465177; doi:10.3389/fgene.2022.931222)
Supplement: Supplementary file 1 [file DataSheet1.ZIP › Supplementary Material.docx]

**Supplementary Figure 1. Clinical correlation analysis of *CENP-A* expression with clinicopathologic features in low-grade gliomas according to TCGA dataset. (A)** Primary therapy outcome, **(B)** Age, **(C)** IDH status, **(D)** 1p/19q co-deletion.

**Supplementary Figure 2. Clinical correlation analysis of *CENP-A* expression with clinicopathologic features in** **CGGA dataset. (A)** WHO grade, **(B)** Age, **(C)** Gender, **(D)** IDH mutation status, **(E)** 1p/19q co-deletion status, and **(F)** IDH mutation status & 1p/19q co−deletion status.

**Supplementary Figure 3. Impact of CENP-A on overall survival probability of glioma patients with different WHO grades in CGGA dataset. (A)** All WHO grade, **(B)** WHO grade II, **(C)** WHO grade III, **(D)** WHO grade IV.

**Supplementary Figure 4. Impact of CENP-A on immune infiltration in glioma with different WHO grades. (A-D)** The correlation between CENP-A expression levels and the enrichment levels of Th2 and pDC cells in grade 2/3 glioma, and **(E-H)** in grade 4 glioma.

**Supplementary Table 1.** Statistical description of ROC curve.

| Group 1 | Group2 | Patient Number | Minimum value | Maximum value | Median | Interquartile range (IQR) | Mean | Standard deviation (SD) | Standard error (SE) |
| --- | --- | --- | --- | --- | --- | --- | --- | --- | --- |
| CENPA | Normal | 1157 | 0.000 | 2.281 | 0.098 | 0.176 | 0.148 | 0.217 | 0.006 |
| CENPA | Tumor | 689 | 0.029 | 5.229 | 1.299 | 2.257 | 1.759 | 1.323 | 0.050 |

**Supplementary Table 2.** AUC results table.

| Predictor variable | Outcome prediction | AUC | CI |
| --- | --- | --- | --- |
| CENPA | Tumor vs Normal | 0.960 | 0.952-0.969 |

**Supplementary Table 3.** Partial ROC information and data when CENPA is set to the optimal cut-off value.

| Predictor variable | Cut-off value | Sensitivity | Specificity | Positive predictive value | Negative predictive value | Youden index |
| --- | --- | --- | --- | --- | --- | --- |
| CENPA | 0.350 | 0.907 | 0.899 | 0.842 | 0.942 | 0.806 |
