# Supplementary figures and images for: CENP-A is a potential prognostic biomarker and correlated with immune infiltration levels in glioma patients
Source: Front Genet. 2022 Aug 29;13:931222. doi: 10.3389/fgene.2022.931222 (PMC9465177; doi:10.3389/fgene.2022.931222)

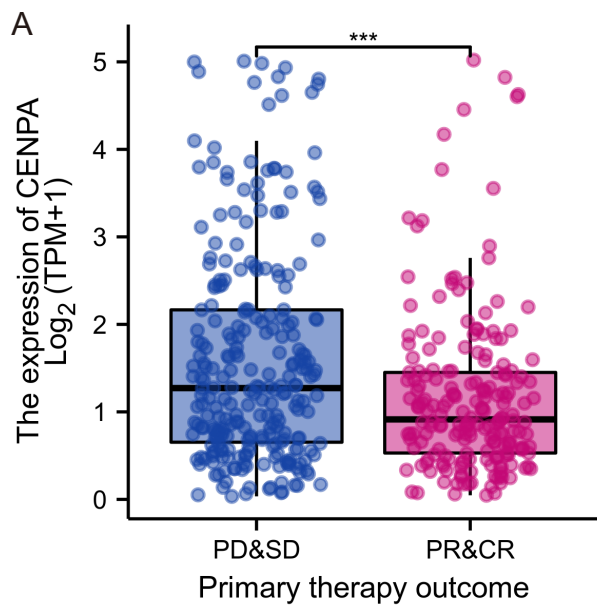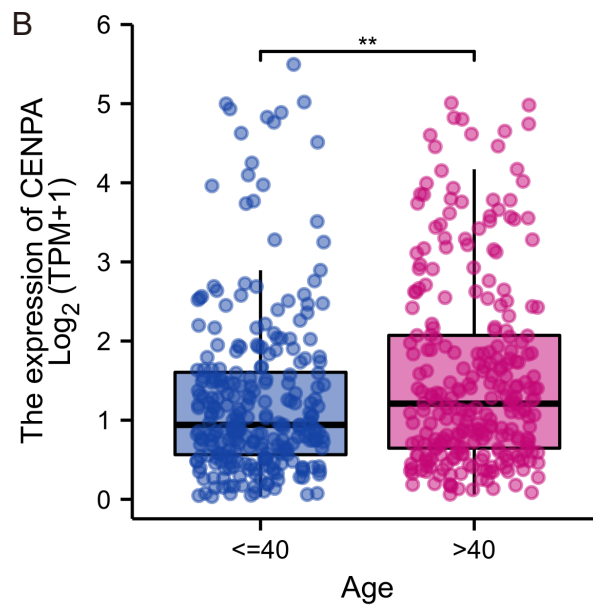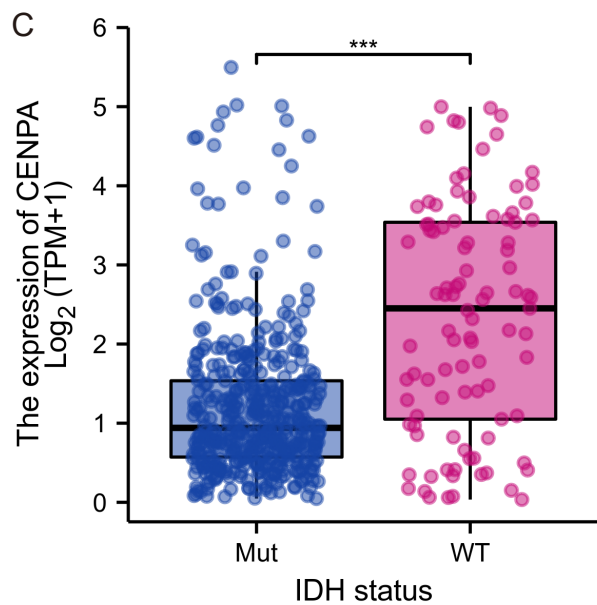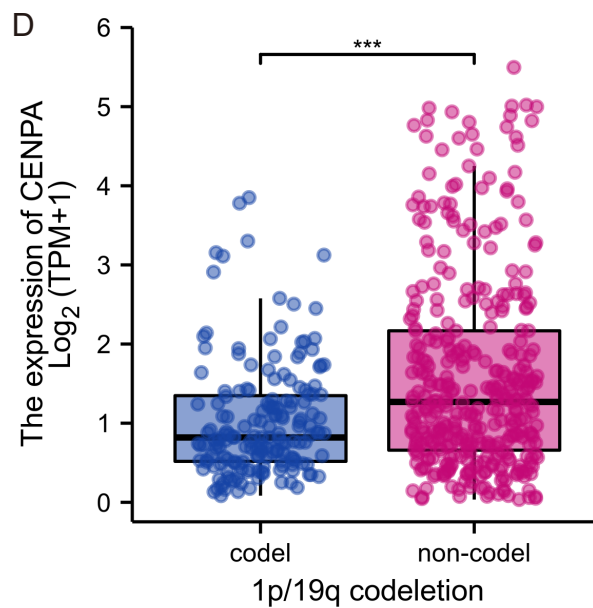

Supplement: Supplementary file 1 [file DataSheet1.ZIP › Supplementary Figure 1.pdf]

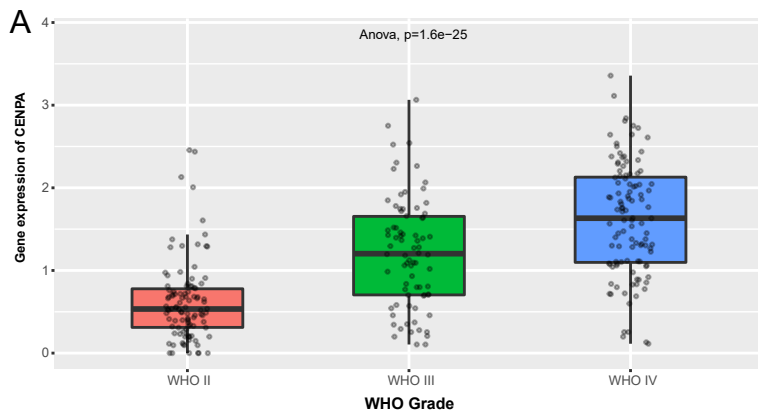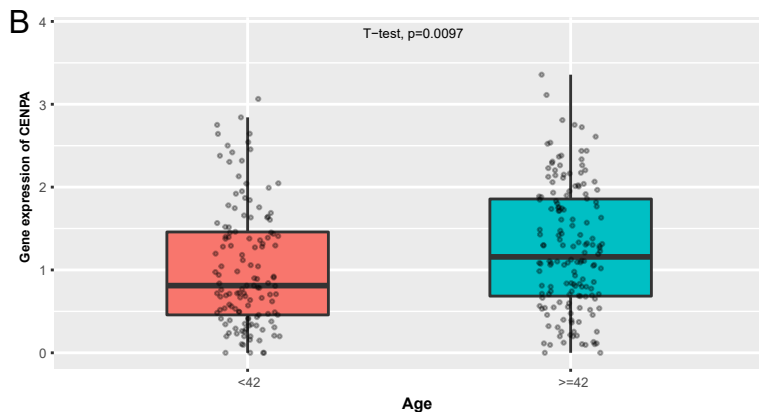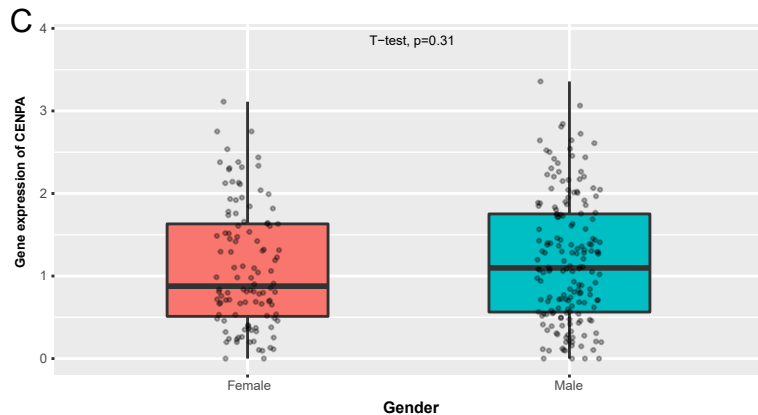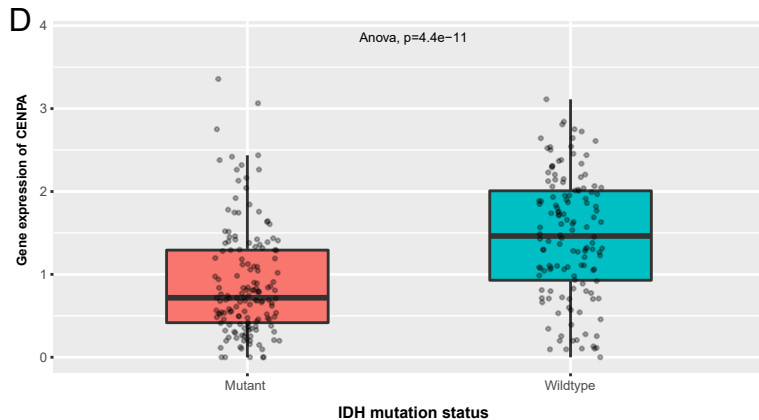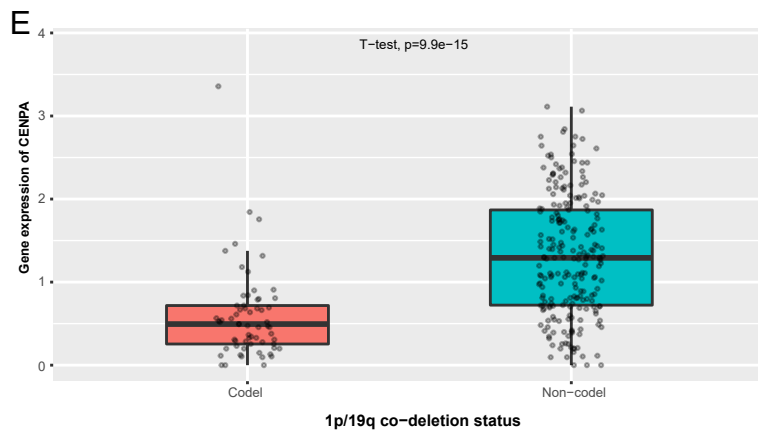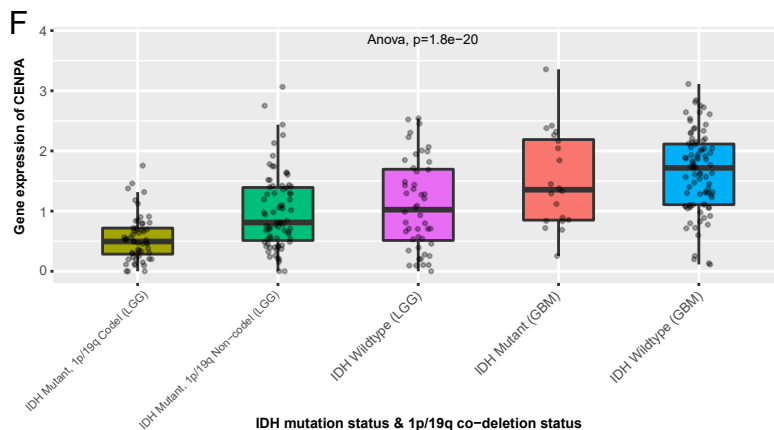

Supplement: Supplementary file 1 [file DataSheet1.ZIP › Supplementary Figure 2.pdf]

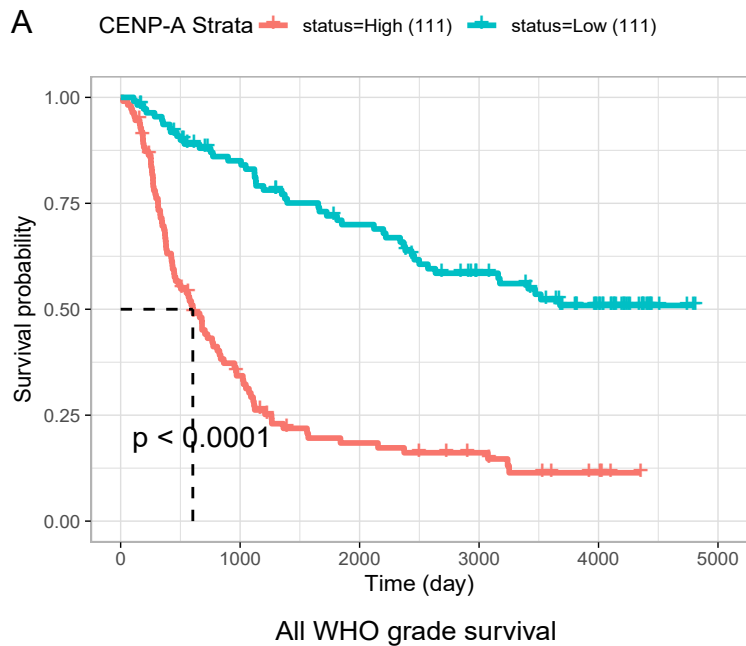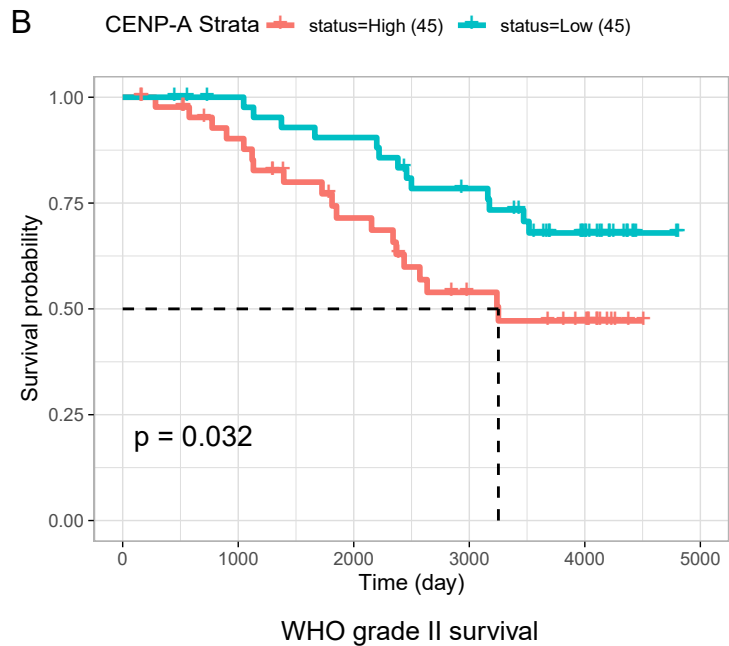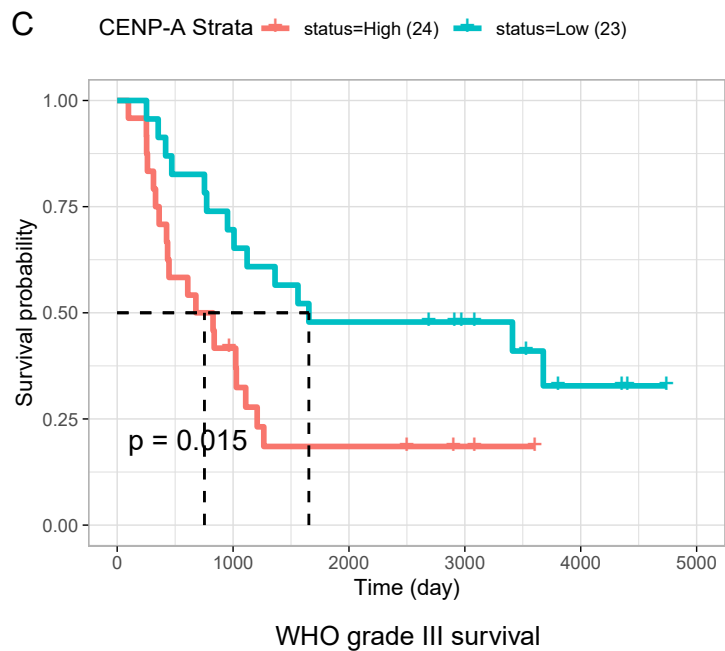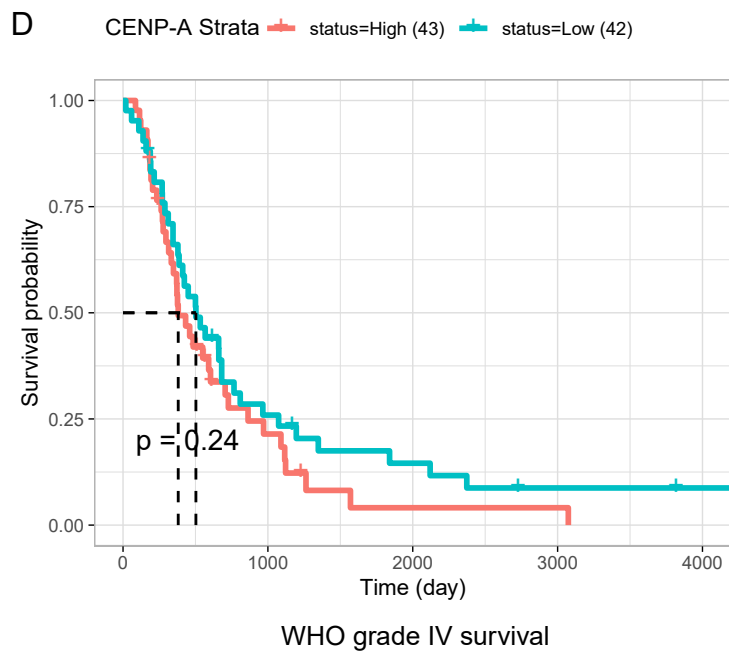

Supplement: Supplementary file 1 [file DataSheet1.ZIP › Supplementary Figure 3.pdf]

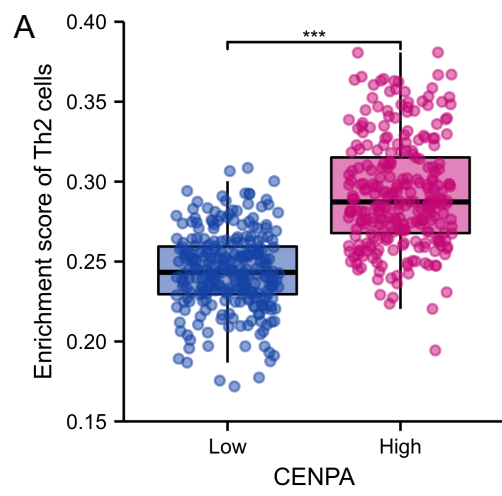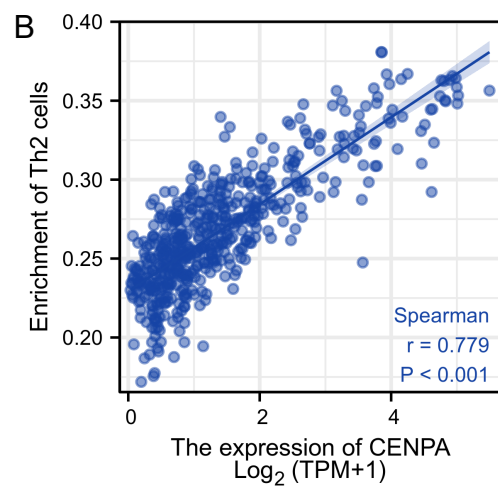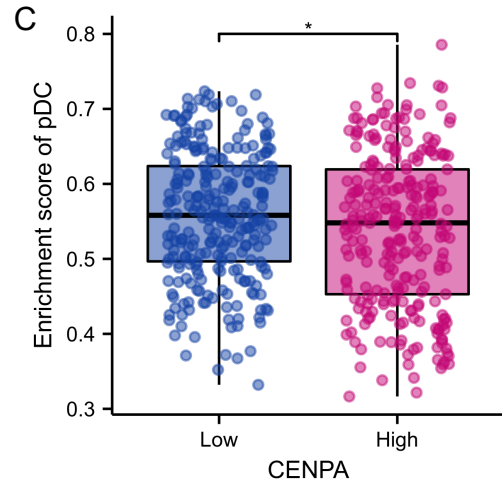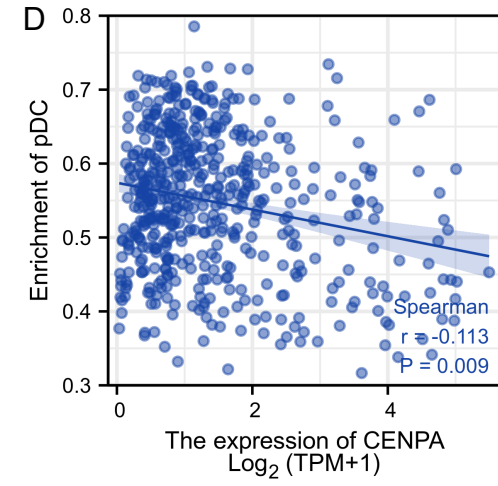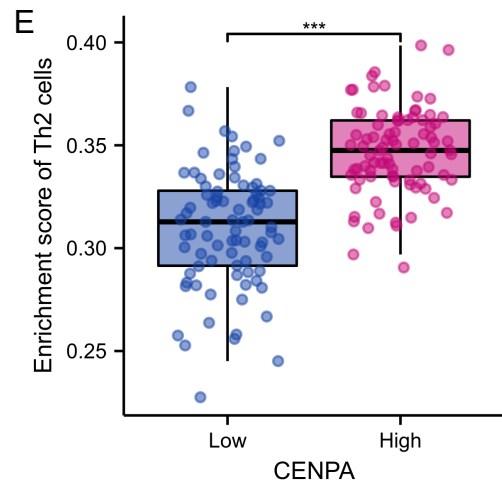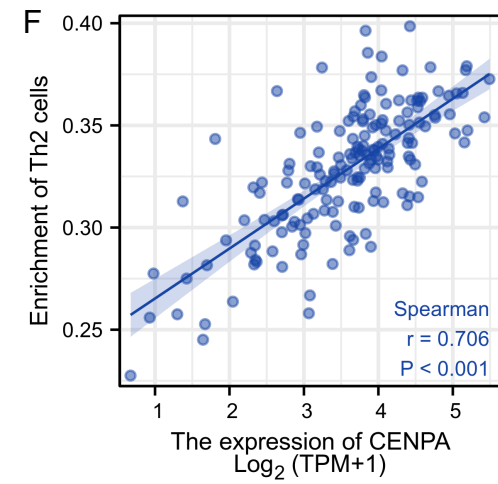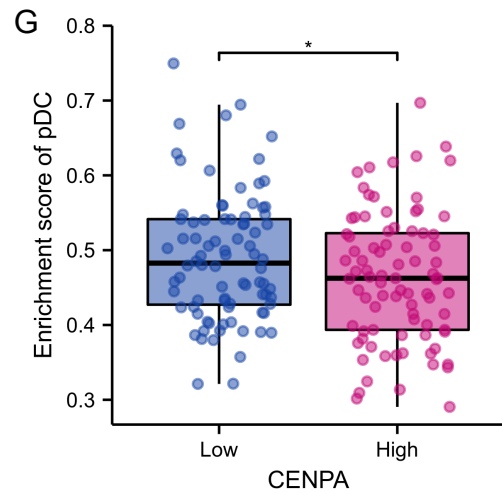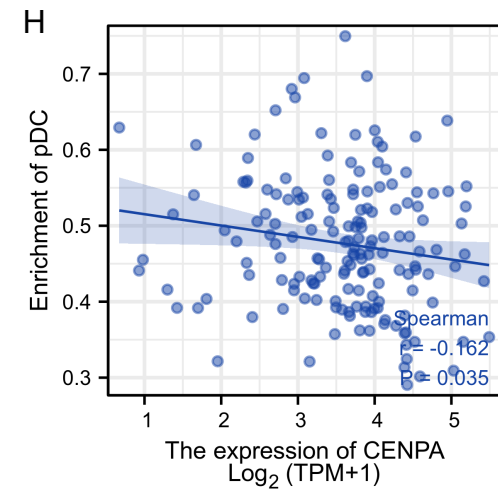

Supplement: Supplementary file 1 [file DataSheet1.ZIP › Supplementary Figure 4.pdf]
